# Top research priorities for preterm birth: results of a prioritisation partnership between people affected by preterm birth and healthcare professionals

**DOI:** 10.1186/s12884-019-2654-3

**Published:** 2019-12-30

**Authors:** Sandy Oliver, Seilin Uhm, Lelia Duley, Sally Crowe, Anna L. David, Catherine P. James, Zoe Chivers, Gill Gyte, Chris Gale, Mark Turner, Bev Chambers, Irene Dowling, Jenny McNeill, Fiona Alderdice, Andrew Shennan, Sanjeev Deshpande

**Affiliations:** 1 Social Science Research Unit, UCL Institute of Education, 18 Woburn Square, London, WH10NR UK; 2 Nottingham Clinical Trials Unit, University of Nottingham, Nottingham, UK; 3 Crowe Associates Ltd, Thame, UK; 4 Institute for Women’s Health, University College London, 86-96 Chenies Mews, London, WC1E 6HX UK; 5 Bliss, London., UK; 6 National Childbirth Trust (NCT), 30 Euston Square, London, NW1 2FB UK; 7 Neonatal Medicine, School of Public Health, Faculty of Medicine, Imperial College London, Chelsea and Westminster Hospital campus, London, SW10 9NH UK; 8 Institute of Translational Medicine, University of Liverpool, Liverpool, UK; 9 London, UK; 10., Ireland; 11 School of Nursing & Midwifery, Queen’s University Belfast, Medical Biology Centre, Belfast, BT9 7BL UK; 12 National Perinatal Epidemiology Unit, Nuffield Department of Population Health, University of Oxford, Old Road Campus, Headington, Oxford, OX3 7LF UK; 13 Kings College London, St. Thomas Hospital, London, SE1 7EH UK; 14 Sanjeev Deshpande, Princess Royal Hospital, Apley Castle, Grainger Drive, Telford, TF1 6TF UK

## Abstract

**Background:**

We report a process to identify and prioritise research questions in preterm birth that are most important to people affected by preterm birth and healthcare practitioners in the United Kingdom and Republic of Ireland.

**Methods:**

Using consensus development methods established by the James Lind Alliance, unanswered research questions were identified using an online survey, a paper survey distributed in NHS preterm birth clinics and neonatal units, and through searching published systematic reviews and guidelines. Prioritisation of these questions was by online voting, with paper copies at the same NHS clinics and units, followed by a decision-making workshop of people affected by preterm birth and healthcare professionals.

**Results:**

Overall 26 organisations participated. Three hundred and eighty six people responded to the survey, and 636 systematic reviews and 12 clinical guidelines were inspected for research recommendations. From this, a list of 122 uncertainties about the effects of treatment was collated: 70 from the survey, 28 from systematic reviews, and 24 from guidelines. After removing 18 duplicates, the 104 remaining questions went to a public online vote on the top 10. Five hundred and seven people voted; 231 (45%) people affected by preterm birth, 216 (43%) health professionals, and 55 (11%) affected by preterm birth who were also a health professional. Although the top priority was the same for all types of voter, there was variation in how other questions were ranked.

Following review by the Steering Group, the top 30 questions were then taken to the prioritisation workshop. A list of top 15 questions was agreed, but with some clear differences in priorities between people affected by preterm birth and healthcare professionals.

**Conclusions:**

These research questions prioritised by a partnership process between service users and healthcare professionals should inform the decisions of those who plan to fund research. Priorities of people affected by preterm birth were sometimes different from those of healthcare professionals, and future priority setting partnerships should consider reporting these separately, as well as in total.

## Background

Preterm birth has major impacts on survival, quality of life, psychosocial and emotional stress on the family, and costs for health services [1]. Improving outcome for these vulnerable babies and their families is a priority, and prioritising research questions is advocated as a pathway to achieve this [2, 3].

Traditionally the research agenda has been determined primarily by researchers, either in academia or industry, who have used processes for priority setting that lack transparency [4, 5]. This has contributed to a mismatch between the available research evidence and the research preferences of patients and members of the public, and of clinicians [6, 7]. Often, research does not address the questions about treatments that are of greatest importance to patients, their carers and practising clinicians [5, 8]. The James Lind Alliance has developed methods for establishing priority setting partnerships between patient organisations and clinician organisations, which then identify and prioritise treatment uncertainties in order to inform publicly funded research [9, 10]. These methods have been used for a range of health conditions [11–17].

We report the outcomes of a process to identify and prioritise research questions in preterm birth that are most important to people affected by preterm birth and healthcare practitioners in the United Kingdom and Ireland using methods established by the James Lind Alliance [18]. This partnership differed from previous priority setting partnerships supported by the James Lind Alliance in that pregnancy is not an illness or disease, and that it involves at least two people (mother and child); in addition preterm birth can have life-long consequences for them, their families and for the health services and society. Our aim was first to identify unanswered questions about the prevention and treatment of preterm birth from people affected by preterm birth, clinicians and researchers. Then to prioritise those questions that people affected by preterm birth and clinicians agree are the most important.

## Methods

**Fig. 1:**
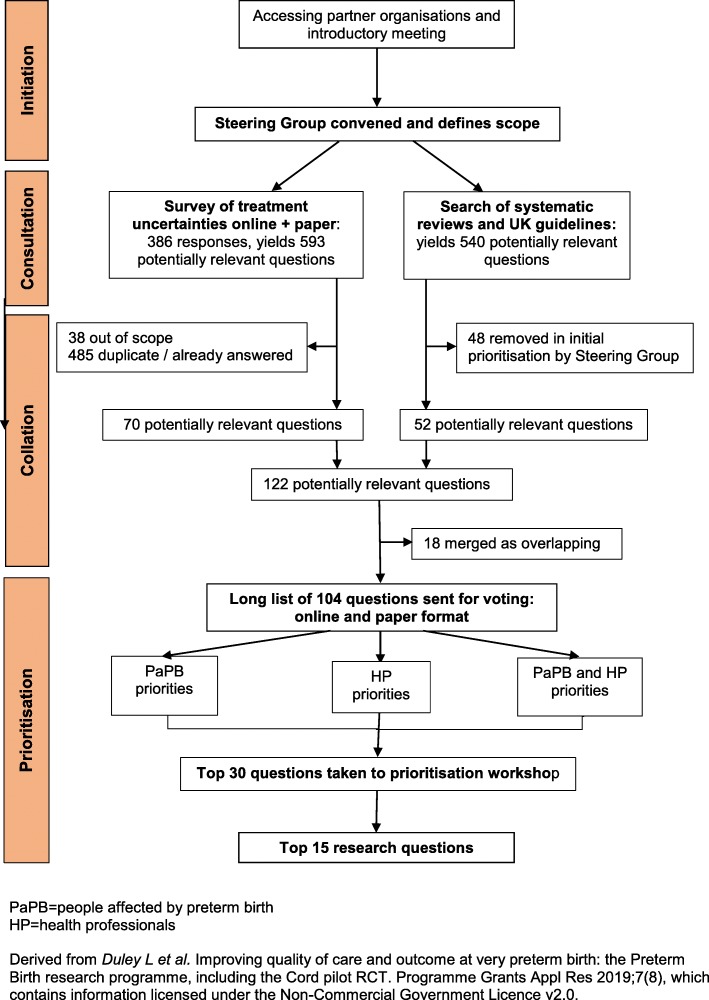
Flow chart of the JLA Preterm Birth Priority Setting Partnership

The Preterm Birth Priority Setting Partnership was convened in November 2011, following an introductory meeting in July 2011. The partnership followed the four stages of the James Lind Alliance process (see Fig. 1) [9].

### Initiation

Organisations whose areas of interest included preterm birth were informed about the priority setting partnership and invited to participate in, or contribute to, the introductory workshop. Those who then joined the partnership are listed in Box 1. All participating organisations were asked to complete a declaration of interests, including disclosure of relationships with the pharmaceutical or medical devices industry. Subsequently a Steering Group was convened, with members of participating organisations who volunteered to take on this role. This group was chaired by a representative from the James Lind Alliance (SC).

At the introductory workshop it was clear that many participants felt the scope of the partnership should be wider than was initially envisaged. Additional topics proposed for inclusion in the scope were uncertainties about the causes of preterm birth, about the prognosis following being born preterm, and about treatments long before birth. As widening the scope too far would risk leaving the prioritisation unachievable within a reasonable time frame and the existing resources, the Steering Group decided the scope would be restricted to uncertainties about treatments, to interventions during pregnancy and around the time of birth or shortly afterwards (taken up to the time of hospital discharge for the baby after birth).

### Consultation to gather research questions (treatment uncertainties)

Research questions were gathered from people affected by preterm birth, clinicians and researchers, using methods developed by the James Lind Alliance [10]. First, a survey was distributed on-line, including through partner organisations, to ask for suggestions about preterm birth experiences, services or treatments which needed to be researched, and why the research would be important (see Additional file 1 for paper version of this survey). Respondents were asked to say if they were people with personal or family experiences of preterm birth, and/or if they were a health professional.

At an interim review of demographic data about home ownership and ethnicity from this survey there was concern that the respondents were not representative of the population at risk of preterm birth. To try and access a more high risk group, paper copies of the survey (see Additional file 1) were distributed at high risk specialist prematurity antenatal clinics at two tertiary level hospitals (University College London Hospital and Queen’s Medical Centre Nottingham), and to parents visiting their babies in three level 3 neonatal intensive care units (University College London Hospital and Chelsea and Westminster Hospital, London; Liverpool Women’s Hospital) between March and December 2012. The survey closing date was extended to allow time to implement these changes. Respondents were invited to provide an email address to be notified about voting to prioritise the questions.

In addition, research questions were identified from systematic reviews of existing research and from national UK clinical guidelines (see Additional file 2).

### Collation - checking and combining research questions

With support from an independent information specialist, submissions from the survey were formatted into research questions, which were checked against existing reviews and guidelines. Those already answered were removed. The remaining research questions were screened by the Steering Group, to remove those answered by a subsequent randomised trial or for which a large randomised trial was in progress, and those that were out of scope or unclear, and to combine similar research questions. This left the final long list of unanswered research questions which was sorted into similar categories, ordered chronologically from before pregnancy to hospital discharge following birth.

### Prioritisation of the research questions

Prioritisation was by a two-stage process using a modified Delphi with individual voting, followed by a face-to-face workshop using nominal group technique [10]. First, the long list of unanswered research questions was made available online for public voting (from September to December 2013), with paper copies distributed to the same high risk antenatal clinics and neonatal units. Respondents were asked to pick the 10 they considered most important. Overall results and results by stakeholder group (people affected by preterm birth, health professional) were reviewed by the Steering Group to remove remaining repetition or overlap between questions. The final shortlist of 30 unanswered research questions to go forward to the prioritisation workshop was then agreed.

The aims of the prioritisation workshop were to agree a ranking for the short list, including the ‘top10’, and to consider next steps to ensure that the priorities are taken forward for research funding. Participants were invited from across the partnership, and included representatives from organisations representing both people affected by preterm birth and clinicians, parents of babies born preterm, and adults who were born preterm. Prior to the workshop, participants were sent the shortlist of unanswered research questions.

At the workshop (held in January 2014), after an introductory session participants were assigned to one of four small groups, each with a facilitator, to discuss ranking for each uncertainty. Groups were pre-specified in advance to include a mix of parents, people born preterm, clinicians and other health professionals. The groups were provided with a set of 30 large cards, each printed with one shortlisted research question. On the reverse were examples of wording from the original submissions, and a breakdown of how people affected by preterm birth and healthcare professionals had scored that question in their voting. Following discussion, these cards were placed in ranked order. Over the lunchtime break, rankings from the four groups were aggregated into a single ranking order. These aggregate rankings were presented at a plenary session, to demonstrate where there was existing consensus between groups, and where there were differences. Participants were then reconvened into three small groups, again pre-planned so each had a new mix of participants and retained a balance of backgrounds, to discuss the aggregate ranking. Similar processes were used as in the earlier small groups, with the aim of agreeing the top ten research questions and ranking all 30 questions. Aggregated ranking from the three small groups was taken to a final plenary session, with the 30 cards laid out on the floor in ranked order. Participants then debated and agreed the final ranking.

## Results

Forty two organisations were approached and invited to participate in the priority setting partnership (see Additional file 4); of these 25 accepted and joined the partnership (see Table 1). Ten organisations were represented on the Steering Group; four representing those affected by preterm birth, and six representing health professionals (obstetricians and neonatologists). Some Steering Group members were parents of infants born preterm, or had themselves been born preterm. The group also included four non-voting members: two researchers who co-ordinated the prioritisation partnership, one a clinical academic with a background in obstetrics and the other with expertise in public engagement in research; one charity representative, and one PhD student.

**Table 1:** Partner organisations

Organisations representing people affected by preterm birth	Both service users’ and clinicians’ organisations	Clinicians’ organisations
• Action on Pre-eclampsia • Bliss, the special care baby charity^a^ • Irish Premature Babies^a^ • Multiple Births Foundation • Cleft Lip and Palate Association • Irish Neonatal Health Alliance^a^ • National Childbirth Trust^a^ • Tiny Life^a^	• Children’s Trust • Tommy’s	• Association of Paediatric Anaesthetists of Great Britain and Ireland • British Academy of Childhood Disability^a^ • British Association of Paediatric Surgeons • British Association of Perinatal Medicine • British Paediatric Pathology Group • British Maternal and Fetal Medicine Society^a^ • Cochrane Neonatal Group • Department of Neonatal Medicine, Imperial College^a^ • MCRN Neonatal Clinical Studies Advisory Group^a^ • Neonatal Nurses Association • Obstetric Anaesthetists Association • Paediatric Intensive Care Society • Royal College of Anaesthetists • Royal College of Obstetrician and Gynaecologists^a^ • UCL Institute of Women’s Health^a^

^a^ Organisations represented on the Steering Group

When the online survey closed it had been accessed by 1076 people, and completed by 349; an additional 37 paper survey forms were completed and returned. Hence a total of 386 people responded of whom 204 (53%) said that they were affected by preterm birth, 107 (28%) that they were health professionals, 43 (11%) that they were both affected by preterm birth and a health care professional, and 32 (8%) did not answer this question (Table 2). Of the 247 respondents affected by preterm birth, most 186 (75%) reported they were parents of a preterm baby, but some were grandparents and other family members.

**Table 2:** Characteristics of respondents to the survey gathering research questions, and to voting about priorities

	Gathering research questions *n* = 386		Voting about priorities *n* = 507	
Type of respondent				
Affected by preterm birth	204	(53%)	231	(45%)
Healthcare professional	107	(28%)	216	(43%)
Affected by preterm birth + healthcare professional	43	(11%)	55	(11%)
Not known	32	(8%)	5	(1%)
Gender^a^				
Female	163	(42%)	422	(83%)
Male	9	(2%)	76	(15%)
Not known	214	(55%)	9	(2%)
Ethnicity^a^				
White	159	(41%)	436	(86%)
Asian	4	(1%)	32	(6%)
Black	9	(2%)	5	(1%)
Chinese	–	–	1	(< 1%)
Mixed	–	–	8	(2%)
Not known	214	(55%)	25	(5%)
Home owner^a^	113	(46%)		

^a^For people affected by preterm birth only, *n* = 247 gathering research questions

The 386 responses contained 593 potential research questions. Submissions were formatted into research questions, with similar submission combined into one question (see Additional file 5), and screened to remove those already answered, out of scope or unclear, (see Additional file 6). Thirty eight submissions were removed as being outside the scope of this process. After merging similar questions and removing those that were fully answered, 70 unanswered questions were left from the survey.

The search of systematic reviews and clinical guidelines identified 540 potentially relevant questions. As there was such a large number, the Steering Group agreed a process to prioritise which would go forward to the next stage. Each member was asked to select the 60 questions from systematic reviews they considered to be most relevant and important. They then brought their list of 60 to a face-to-face meeting at which questions were only considered as potential priorities for the voting stage if they were supported by three or more members. This resulted in 28 questions from systematic reviews and 24 from clinical guidelines remaining in the process. Overall there were then 122 questions; as 18 of these overlapped with other questions, they were merged to give a final ‘long list’ of 104 unanswered research questions (see Additional file 3).

The 104 questions on the long list were sent for an online public vote, with paper copies distributed to the same high risk antenatal clinics and neonatal units. Overall 507 people voted (448 online and 59 on paper); 231 (45%) said they had been affected by preterm birth, 216 (43%) that they were a health professional, and 55 (11%) that they were affected by preterm birth and also a health professional (Table 2). Type of respondent was not known for 5 (1%) voters. Of the 271 who said they were a health professional (including those who had been affected by preterm birth themselves), 85 said they were an obstetrician, 51 a nurse, 44 a neonatologist, 24 a midwife, 4 a general practitioner, 32 were other health professionals and 31 preferred not to say. Of those who voted, 512 (87%) reported their ethnicity as white, and ethnicity was not known for 8 (2%). Responses were received from the four nations within the United Kingdom, and from the Republic of Ireland.

For public voting, the top priority (which treatments (including diagnostic tests) are most effective to predict or prevent preterm birth?) was the same for all three types of respondent (Table 3), but there was considerable variation in how other questions were ranked. Several questions were in the overall top 10 for only one type of voter. Questions ranked 1–40 in the public vote were reviewed by the Steering Group, taking into account the voting preferences of people affected by preterm birth and the overall balance of the topics. Four questions were removed: one had already been answered, one was being addressed by an ongoing trial, and two were merged with another broader question (all three being about infant feeding). A shortlist of the top 30 questions was then taken forward to the prioritisation workshop (Table 4).

**Table 3:** For the public vote: top 10 research questions by type of voter (those in italics cells were in the top 10 for one type of voter only)

Type of respondent for public vote			
	Service user	Health professional	Service user & health professional
1	Which treatments (including diagnostic tests) are most effective to predict or prevent preterm birth?	Which treatments (including diagnostic tests) are most effective to predict or prevent preterm birth?	Which treatments (including diagnostic tests) are most effective to predict or prevent preterm birth?
2	What treatments can predict reliably the likelihood of subsequent infants being preterm?	What is the optimum milk feeding regimen, for preterm infants, including quantity and speed of feeding and use of donor and formula milks?	What is the optimum milk feeding regimen, for preterm infants, including quantity and speed of feeding and use of donor and formula milks?
3	How do stress, trauma and physical workload contribute to the risk of preterm birth, are there effective ways to reduce those risks and does modifying those risks alter outcome?	*Which treatments are most effective to prevent necrotising enterocolitis in preterm infants?*	How do stress, trauma and physical workload contribute to the risk of preterm birth, are there effective ways to reduce those risks and does modifying those risks alter outcome?
4	What should be included in packages of care to support parents and families / carers when a premature baby is discharged from hospital?	Which treatments are most effective to prevent pre-eclampsia (for example, progesterone, calcium, garlic etc)?^a^	What should be included in packages of care to support parents and families / carers when a premature baby is discharged from hospital?
5	What is the optimum milk feeding regimen, for preterm infants, including quantity and speed of feeding and use of donor and formula milks?	Which treatments are effective in preventing spontaneous preterm birth in women with twin and triplet pregnancies, especially in those at high risk of preterm birth?^a^	*What type of support is most effective at improving breastfeeding in NICU / SCBU / feeding clinics?*
6	Which treatments are most effective to prevent pre-eclampsia (for example, progesterone, calcium, garlic etc)?	*What methods are most effective to predict risk of preterm birth in order to allocate service provision?*^a^	What treatments can predict reliably the likelihood of subsequent infants being preterm?^b^
7	How can infection in preterm infants be better prevented?^d^	*Is routine transvaginal scanning during pregnancy to detect short cervical length, and treatment, cost effective?*^a^	Is screening in the first trimester effective to help prevent preterm birth?^b^
8	Can screening of the placenta be effective to detect placenta abnormalities associated with preterm birth?^d^	Is screening in the first trimester effective to help prevent preterm birth?^c^	Which treatments are most effective to prevent pre-eclampsia (for example, progesterone, calcium, garlic etc)?
9	*What is the best way to judge whether a baby is feeling pain (for example, by their face, behaviours or brain activities)?*	Does screening and treatment for Group B Streptococcus help to prevent preterm birth and neonatal morbidity and mortality?^c^	*Do preterm babies have better outcomes if their parents have roomed in?*
10	Is screening in the first trimester effective to help prevent preterm birth?	*What is the best time to clamp the umbilical cord for preterm babies?*	How can infection in preterm infants be better prevented?

^a, b, c, d^ these questions had the same number of votes within this type of voter category

**Table 4:** For the prioritisation workshop: final ranking for the 29 research questions (two questions were merged due to overlap) and ranking overall ranking from the public vote

Ranking following the prioritisation workshop		Ranking from public vote
1	Which treatments (including diagnostic tests) are most effective to predict or prevent preterm birth?	1
2	How can infection in preterm infants be better prevented?	8
3	Which interventions are most effective *to prevent* necrotising enterocolitis in preterm infants?	9
4	What is the best treatment for life-threatening lung damage in preterm infants?	20
5	What should be included in packages of care to support parents and families / carers when a premature baby is discharged from hospital?	6
6	What is the optimum milk feeding strategy and guidance (including quantity and speed of feeding and use of donor and formula milk) for the best long-term outcomes of premature babies?	2
7	What is the best way to judge whether a baby is feeling pain (for example, by their face, behaviours or brain activities)?	14
8	Which treatments are most effective to prevent early onset pre-eclampsia?	5
9^a^	What emotional and practical support improves attachment and bonding, and does the provision of such support improve outcomes for premature babies and their families?	25 / 28^a^
10	Which treatments are most effective for premature rupture of membranes?	16
11	What is the best time to clamp the umbilical cord for preterm babies?	19
12	What type of support is most effective at improving breastfeeding in NICU/SCBU/feeding clinics?	12
13	Which treatments are most effective *to treat* necrotising entercolitis in preterm infants?	22
14	Does specialist antenatal care for women at risk of preterm birth improve outcomes for mother and baby?	11
15	What are the best ways to optimise the environment (such as light and noise) in order to improve outcomes for premature babies?	26
16	Is screening in the first trimester effective to help prevent preterm birth?	7
17	Which treatments are effective in preventing spontaneous preterm birth in women with twin and triplet pregnancies, especially in those at high risk of preterm birth?	10
18	How do stress, trauma and physical workload contribute to the risk of preterm birth, are there effective ways to reduce those risks and does modifying those risks alter outcome?	3
19	Is routine transvaginal scanning during pregnancy to detect short cervical length, and treatment, cost effective?	18
20	What guidance and information is most useful for parents at risk of having preterm infants?	21
21	Does screening and treatment for Group B Streptococcus help to prevent preterm birth and neonatal morbidity and mortality?	15
22	What is the impact of length of orogastric / nasogastric feeding and reflux on early feeding development in preterm infants?	24
23	What methods are most effective to predict risk of preterm birth in order to allocate service provision?	17
24	Can screening of the placenta be effective to detect placenta abnormalities associated with preterm birth?	13
25	What is the best way to encourage Kangaroo Mother Care more by staff in NICU for parents?	23
26	What treatments can predict reliably the likelihood of subsequent infants being preterm?	4
27	Do parents of preterm infants benefit from an open approach to notes and ward rounds?	27
28	Do preterm babies have better outcomes if their parents have roomed in?	29
29	Which lifestyle changes including gym, bed rest, posture and sexual intercourse are effective to minimise the risk of preterm birth?	30

^a^two original questions merged

The workshop to prioritise these 30 questions was attended by 34 participants; 13 parents or adults who had been born preterm and 21 health professions (neonatology, obstetrics, midwifery, speech therapy and psychology). Several of the health professionals also had personal experience of preterm birth. In addition, there were four facilitators (two from the James Lind Alliance and two non-voting members of the Steering Group), five observers (one from the James Lind Alliance, one from a research funding organisation in Canada, one from the Institute of Education University of London, and two who were non-voting members of the Steering Group).

During the prioritisation workshop, two questions were merged as it was agreed they overlapped, and the wording of a few others was modified for clarification. Following the first round of small group discussion, there was considerable variation in the top priorities between the four groups. Following the second round of small group discussion there was agreement about the top few priorities. During the final plenary discussion about the aggregated ranking there was consensus about the top seven questions, less consensus about the next three, and disagreement about those ranked as between 10 and 20. As it was not possible to achieve consensus about the top 10 questions within the timeframe, a proposal to expand this to a top 15 was agreed. Consensus about the top 15 was then achieved (Table 4). This top 15 had some significant differences to the ranking following public voting. The most noticeable was two questions ranked 18 (How do stress, trauma and physical workload contribute to the risk of preterm birth, are there effective ways to reduce those risks and does modifying those risks alter outcome?) and 26 (What treatments can predict reliably the likelihood of subsequent infants being preterm?) at the workshop were ranked 3 and 4 respectively in the overall public vote, and 2 and 3 by service users in the public vote (Table 3).

## Discussion

The unanswered research questions relevant to preterm birth identified during this process were prioritised in the United Kingdom and Republic of Ireland by people affected by preterm birth (parents, grandparents, adults who were born preterm, and others affected by preterm birth), by a range of health professionals, and by people who were both personally affected by preterm birth and a health professional. To our knowledge this is the first such process in preterm birth. People affected by preterm birth and health professionals had many shared priorities, but our process demonstrates that on some questions they have different perspectives. Priorities may also change over time and in different settings, Hence, although the top research priorities from this process should be considered by those who plan and fund research in this area, the full list of 104 unanswered questions is also relevant to decision-making about research funding. This is particularly true if we wish to make research more relevant to those whose lives have been affected by preterm birth, and the healthcare workers who care for them.

While several of the top priorities for research are broad topics already well recognised as important, such as what is the optimum milk feeding regimen for preterm infants and prevention of infection, others are indicative of areas previously underrepresented in research; for example packages of care to support families after discharge, and what is the role of stress, trauma and physical workload in the risk of preterm birth, and are there effective ways to reduce this risk and does this influence outcome. This is in keeping with findings from previous James Lind Alliance partnerships, which suggests and highlights the value of partnership and shared decision making with an inclusive stakeholder group with balanced representation of service users and clinicians [7].

In line with the literature on consensus development [19], the strengths of this Preterm Birth Priority Setting Partnership include the large numbers of participants in the process, the range of stakeholders involved, the formality of the processes, the use of facilitators for face-to-face debate to ensure that all options were discussed and all participants had a chance to voice their views, providing feedback and repeating the judgment, and ensuring that judgements were made confidentially. The first three features applied to both the consultation and the workshop; the last applied only to the consultation. The change in priorities between the survey and the workshop deserves further investigation. Although the choice of individuals within the professional groups represented is unlikely to have made a difference to the priorities, [20] difference in status across workshop participants may have [19].

Preterm birth is associated with factors such as lower socio-economic status, ethnicity (such as African origin), and maternal age (being lower than 18 years or above 35 years) [21]. Despite implementing strategies to reach a more representative population, our respondents remained primarily white and with a relatively high proportion of homeowners, hence not representative of the population affected by preterm birth. This could limit generalisablity of these priorities to other populations. A wide range of relevant health professionals participated in the public voting, including neonatologists, obstetricians, neonatal nurses, midwives, speech and language therapists, psychologists and general practitioners; strengthening generalisablity.

Maintaining balanced representation between people affected by preterm birth and the different groups of health professionals for the final prioritisation workshop was challenging. This may have had implications for the final decisions, as happens in guideline development, where consensus development research concludes that differences in how groups are constituted (but not individual members) leads to different decisions [22]. At our workshop differences in priorities between the various professional groups contributed to the difficulty in achieving consensus for a top 10 list, and to the two ‘lost priorities’ which although ranked in the top 5 at the public vote were not included in the final top 15. The difficulty in agreeing a top 10 underlines the complexity of priority setting for research, particularly for topics such as preterm birth, which involve mother and baby, as well as their wider family. This complexity, and the differing priorities of different stakeholders, make it important to publicise the top 30 list, and the full long list of 104 questions, as well as the top 15 priorities [23].

Large changes in ranking following the public vote and the final prioritisation appeared to be related to difficulty in the perspective of people affected by preterm birth being heard in the large group session, and an imbalance between the different priorities of two key types of health professional (neonatologists and obstetricians). This was further complicated by fewer obstetricians than expected attending the workshop, and by some of the healthcare professionals also being researchers. Another element of our work, reported in detail elsewhere, was a nested observational study of how service users and healthcare professionals interact when making collective decisions about research priorities [24]. This suggested that health care professionals and service users tended to use different pathways for persuasion in a group discussion, and communication patterns depended on the stage of group development. The Steering Group had worked together for some time, and when new participants joined for the workshop communication patterns returned to an earlier stage. This may have influenced quality of the consensus.

Reporting of the process for prioritisation is therefore important for transparency, and to identify ways to improve it. Future prioritisation processes, particularly those with a similar wide range of healthcare professionals, should endeavour to anticipate potential different perspectives and mitigate any imbalance where possible, and should report voting separately by ‘service users’ and healthcare professionals. Similarly, whilst it may be appropriate to include healthcare professionals who are also researchers in prioritisation, this potential conflict of interest should be declared and taken into account.

This priority setting was limited to the United Kingdom and Ireland, and is therefore most readily generalisable to settings with a similar population and health system. Previous research prioritisation processes for preterm birth [3, 25] did not include people affected by preterm birth and were for low and middle income settings. The most recent neonatal prioritisation exercise in the UK did not include people affected by preterm birth and considered only medicines for neonates [26]. Although unanswered research questions are universal, prioritisation of these questions depends on the local values, context and setting. Nevertheless, there are common priorities across these different settings and our prioritisation process in the UK, such as prevention of preterm birth, postnatal infection and lung damage.

Failure to take account of the views of users of research (i.e. clinicians and the patients who look to them for help) contributes to research waste [27]. James Lind Alliance priority setting partnerships brings together ‘patients, carers and clinicians’ to identify unanswered research questions and to agree a list of the top priorities, (http://www.jla.nihr.ac.uk/about-the-james-lind-alliance/about-psps.htm) which can then shape the health research agenda [12–14]. The aim is to ensure that those who fund health research, and also those who support and conduct research, are aware of what really matters to both patients and clinicians. In our priority setting partnership, people affected by preterm birth and the different groups of health care professionals had different priorities. This underlines the importance of this paper presenting the full list of 30 questions taken forward to the prioritisation workshop, and the respective priorities of people affected by preterm birth and health professionals, as well as the long list of 104 unanswered questions sent out for public voting.

## Conclusions

We present the top 30 unanswered research questions identified and prioritised by the priority setting partnership, along with the full list of 104 questions. These include treatment and prevention as well as how care should be organised and staff training. They should be publicised to the public, to research funders and commissioners, and to those who support and conduct research.

People affected by preterm birth and health professionals sometimes had different priorities. Future priority setting partnerships should consider reporting the priorities of service users and healthcare professionals separately, as well as in total. Those with a wide range of healthcare professionals involved should anticipate potential different perspectives and mitigate any imbalance where possible. Healthcare professionals who are also researchers should declare this potential conflict before participating in prioritisation, so that it can be taken into account.

## Supplementary information

**Additional file 1.** Survey form.

**Additional file 2.** Mapping systematic reviews.

**Additional file 3.** Long list of questions sent for voting.

**Additional file 4.** Organisations invited to participate.

**Additional file 5.** Submissions formatted as research questions.

**Additional file 6.** Reasons for excluding submissions.

## Data Availability

Datasets generated and analysed during the current study are available from the corresponding author on reasonable request.
