# Supplementary material for: Top research priorities for preterm birth: results of a prioritisation partnership between people affected by preterm birth and healthcare professionals
Source: BMC Pregnancy Childbirth. 2019 Dec 30;19:528. doi: 10.1186/s12884-019-2654-3 (PMC6938013; doi:10.1186/s12884-019-2654-3)

- ***Service user** and **health professional organisations** are **working together** to gather the most important ideas for research about **preterm birth and caring for premature babies.** This is called the 'James Lind Alliance (JLA) Preterm Birth Priority Setting Partnership (PSP)'.*
- *All the research questions that need answering will be published in UK Database of Uncertainties about the Effects of Treatments (www.library.nhs.uk/duets).*
- *For more information about this project, please visit here*
- *This survey is also available on-line. Go to http://www.surveymonkey.com/s/prembabies to take part in this way.*

PART ONE – YOUR SUGGESTIONS OR QUESTIONS FOR RESEARCH ABOUT PRETERM BIRTH AND PREMATURE BABIES

You are invited to suggest topics or questions about preterm birth and caring for premature babies. You can make suggestions about the followings or anything else:

- How to investigate the causes of preterm birth?
- Are there better ways of preventing or treating preterm birth or caring for premature babies?
- Whether treatments are safe or effective?
- Family care and support?

Examples for other health conditions:

- *Does listening to babies' hearts when they are three months old find those babies that need special heart care?*
- *Do 'buddies' (or peer support) benefit children with Down's syndrome and their families?*

QUESTION 1 YOUR SUGGESTION OR QUESTION

Is there an experience, service or treatment about preterm birth which needs to be researched? (If you have more than one suggestions, we would like to hear all of them.)

____

____

____

____

____

____

QUESTION 2 [OPTIONAL]

How, or why is your suggestion or question important to you, other people, or for particular reasons?

____

____

____

PART TWO – ABOUT YOU (The following information is helpful us to understand backgrounds of people, who have answered this survey. The information will be collected anonymously for statistical purpose. If you wish you can skip the questions.)

QUESTION 3 Are you______________________?
(Please tick the appropriate option)

- ☐ Service user (parent, family, grandparent, carer etc) – Go to Question 4
- ☐ health care professional– Go to Question8
- ☐ Both service user and health care professional – Go to Question 4
- ☐ Prefer not to say – Go to Question 4

QUESTION 4 (Service users only) Are you_______________?

- ☐ Parent of (a) preterm babies
- ☐ Someone who was born prematurely
- ☐ Grandparent of (a) preterm babies
- ☐ Carer of (a) preterm babies or families with (a) preterm babies
- ☐ Other family member of (a) preterm babies
- ☐ Other - Please specify__________________________________

QUESTION 5 Are you________________?
(Please tick the appropriate option)

- ☐ Female
- ☐ Male

QUESTION6 How would you describe your ethnic group?
(Please tick the appropriate option)

□ White

□ Mixed

□ Asian

□ Black

□ Chinese

□ Other Ethic Group

□ Prefer not to say

QUESTION 7 Can you please describe the housing that you are currently living in? (Please select the closest one)

□ own property (or have mortgage)

□ rented accommodation

□ living with family or relatives

□ Other accommodation (please specify) ______________________________

□ Prefer not to say

QUESTION 8 (Health Care Professionals only)
Please tell us what kind of health care professional you are.

□ Midwife

□ Nurse (Please state specialism, eg Neonatal nurse)___________________________________

□ General practitioner

□ Obstetrician(Please state seniority, eg Consultant, Registrar or SHO) _________________

□ Neonatologist (Please state seniority, eg Consultant, Registrar or SHO) _________________

□ Other Medical doctor or surgeon (Please state specialism)_________________

□ Other - Please specify____

□ Prefer not to say

PART 3 You have completed the survey - thank you for taking part.

The next stage is to prioritise the unanswered questions for research. We will keep you informed via our website (http://eppi.ioe.ac.uk/pretermbirth) and Twitter feed (@PretermBirth).

To get involved, or to find out more, please fill in your contact details below (these will be stored securely and not passed on to anyone else).

Title__________

First Name ____

Family Name____

Address____

____

____

Post Code______________

E-mail address (if applicable)____

Telephone Number____________________________________

Please return the completed survey to Seilin Uhm Social Science Research Unit, Institute of Education, University of London, 18 Woburn Square, London WC1H ONR by Monday 31st of December 2012

For any further questions, please contact Seilin (s.uhm@ioe.ac.uk): Tel: +44(0)20 7612 6532

This survey is also available on-line. Go to http://www.surveymonkey.com/s/prembabies *to take part in this way.* **Thank you for your participation!**

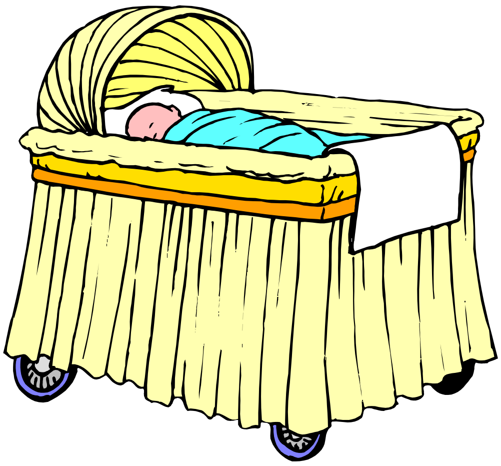

Supplement: Supplementary file 1 — Additional file 1. Survey form. [file 12884_2019_2654_MOESM1_ESM.pdf]
