# Supplementary material for: Top research priorities for preterm birth: results of a prioritisation partnership between people affected by preterm birth and healthcare professionals
Source: BMC Pregnancy Childbirth. 2019 Dec 30;19:528. doi: 10.1186/s12884-019-2654-3 (PMC6938013; doi:10.1186/s12884-019-2654-3)
Supplement: Supplementary file 2 — Additional file 2. Mapping systematic reviews. [file 12884_2019_2654_MOESM2_ESM.docx]

**Additional file 2**

**Mapping systematic reviews**

**Background and research questions**

The Priority Setting Partnership was set up to identify and prioritise the unanswered questions about treatments that they agree are most important to the service users and health care professionals, who are involved in the health condition. We conducted a review of the systematic reviews (mapping) to identify outcomes commonly used to assess the effects of care at the time of preterm birth, and questions of effectiveness that remain unanswered by systematic reviews.

The initial protocol for the mapping was developed to address the following questions:

- What up-to-date systematic review evidence is available about the effects of care at preterm birth?
- What gaps in the evidence are identified by systematic reviews of effects of care at preterm birth?

After comparison with surveys from clinicians and patients, the following questions were added to the protocol to eliminate the duplicates and to identify the ‘true uncertainties’:

- What uncertainties about effects of care expressed by clinicians or service users have up-to-date systematic reviews evidence addressing them?
- What uncertainties about effects of care expressed by clinicians or service users do not have up-to-date systematic review evidence addressing them?

**Scope of the review**

To be included in the mapping, studies needed to meet all of the following inclusion criteria:

- **Intervention**

Eligible studies focus on interventions which aim to improve quality of care and outcomes at preterm birth.

- **Population**

Eligible studies focus on birth at less than 37 weeks gestational age.

- **Research design**

Eligible studies are systematic reviews about care at the time of preterm birth. These may be reviews of effectiveness, observational or qualitative studies.

**Identifying studies**

Search concepts were identified as ‘preterm birth’ AND ‘systematic review’ [Figure 1]. Appropriate key words (search terms including free text and Mesh terms) to retrieve the overlapping area of the two concepts were then explored. The key words were displayed in Table 1 and 2.

**Figure 1** Search concepts

**Table 1** Search terms for Preterm Birth

| **Prematurity** | **Birth** |
| --- | --- |
| **Free text**  very low birth weight  VLBW  extremely low birth weight  ELBW  very low birthweight  extremely low birthweight  Very-low-birth-weight  very low birth-weight  extremely-low-birth-weight  extremely low birth-weight  Preterm  pre-term  Premature  Prematurity  **MeSH Terms**  infant, low birth weight  infant, premature  infant, premature, diseases | **Free text**  Infant  Newborn  Newborns  Infants  Baby  Babies  Birth  neonates  Neonate  **MeSH Terms**  premature birth |

**Table 3** Search terms for systematic review

| **Systematic Review** | | | |
| --- | --- | --- | --- |
| **Free text** | | | **MeSH Terms** |
| **Combinations with “search”**  Literature  Database  Comprehensive  Extensive  Exhaustive  Purposive Representative  Synthesis  Synthesise  Systematic | **Combination**s **with “review”**  Systematic  evidence  Meta regression  meta synthesis  meta- synthesis  meta analysis  metaanalysis  meta-analysis  literature  metanalysis  Metaregression  Meta-regression  Methodological overview  Methodologically overview  pooled analysis  pooled data  Quantitative overview  Effectiveness  Effects  Synthesis  Integrated  Mapping  Methodological  Methodologically  Intervention  Quantitative  research integration | **Other search terms**  Systematic  Overview  Synthesis  meta-analysis  Metaanalysis  meta analysis  Search strategy | review literature as topic |

The search terms were then adjusted, combined and applied to the following sixteen electronic sources: the Cochrane Library, PsycINFO (from American Psychological Association), MEDLINE, EMBASE, British Nursing Index (BNI), Maternity and Infant Care(MIDIRS), Health Management Information Consortium (HMIC), Database of Abstracts of Reviews of Effects (DARE), Turning Research Into Practice (TRIP), the Cumulative Index to Nursing and Allied Health Literature (CINAHL), NICE Compliance Database (NICE), POPLINE (POPulation information onLINE), Information and Knowledge Services of Kings Fund library, The National Perinatal Epidemiology Unit’s website, Joanna Briggs Institute (JBI). Detailed search term combinations with the search stages of each database are described at the end of this document.

Citations from the search outcomes from each database were uploaded into specialist reviewing information management software, EPPI-reviewer. After checking for duplicates, titles and abstracts were screened for eligibility. Citations were excluded hierarchically if they are:

- Not about preterm infants or preterm birth
- About interventions for preventing preterm birth
- About postnatal health care
- Not a review or a protocol for a systematic review
- For other reasons

If reviewers ‘request a second opinion’, the citation was discussed before a decision about eligibility is made.

Four reviewers screened the same 30 citations and compare their findings. After discussing discrepancies, and refining definitions, the exercise were repeated twice more before the remaining citations are screened by reviewers individually. The full text was retrieved for citations potentially eligible after screening. Studies were excluded hierarchically if they are:

- Not about preterm infants or preterm birth (population)
- About interventions for preventing preterm birth
- About postnatal care
- Not a review or review protocol
- Not a review with clear inclusion criteria
- Not a review with clear search strategy (study design)
- Other reasons(e.g. full text not available)

**Describing sets of outcomes**

In the original plan, the focus of each systematic review was described in terms of:

1. Population
   1. Newborns
      1. Age Less than 28 weeks

28 to less than 32 weeks

32 to less than 37 weeks

- - 1. Health conditions
       1. Mortality
          1. Neonatal period (28 days )
          2. Infancy (first year)
       2. Acute Complications
          1. Lungs and respiratory system

Respiratory distress syndrome (RDS)

Bronchoplumonary Dispalsia(BPD) and Chronic Lund Disease(CLD)

Apnea

- - - - 1. Gastrointestinal system (GI)

Feeding intolerance

Necrotising enterocolitis (NEC)

Gastroesophageal reflux (GER)

- - - - 1. Skin
        2. Infections and immune system

Pneumonia

Sepsis

Meningitis

Urinary tract infections

Invasive fungal infections

Disseminated fungal infections

- - - - 1. Cardiovascular system

Heart failure

Patent ductus arteriosus

Hypotension

Apnea/bradycardia

- - - - 1. Hematologic system

Anemia

- - - - 1. Auditory system and hearing
        2. Ophthalmic system and vision
        3. Central nervous system
      1. Neurodevelopmental complication
         1. Motor impairment

Cerebral palsy (CP)

Coordination and motor planning

- - - - 1. Cognitive impairment

Cognitive test scores and mental retardation

School problems

- - - - 1. Visual impairment
        2. Hearing impairment
        3. Behavioural and social-emotional problems
        4. Severity of disability
  1. Mothers
     1. Emotional distress
        1. Supervision of child
        2. The child’s peer relationship
        3. The child’s self-esteem
        4. Impact on the mother’s role in the family due to her absence
     2. Strain and compromised sense of mastery
     3. Health-related Quality of Life (QoL)
  2. Parents
     1. Difficulty in employment
     2. Gender role differences
     3. Parents’ marital relationship
  3. Families
     1. Family stress at different ages
     2. The impact of the child’s difficulties on family routine
     3. Limitation in family social life
     4. Financial burden
     5. Siblings
        1. Decreased attention
     6. Family’s positive interaction
  4. Professional Carers

1. Interventions
   1. (Antenatal interventions)
      1. Vitamin A to the mother
   2. Intrapartum interventions
2. Prophylactic corticosteroid therapy in preterm labour
3. Antibiotics for PROM/PPROM
4. Antibiotics for preterm labour with intact membranes
5. Delayed cord clamping
   1. Postnatal interventions
6. Neonatal resuscitation
7. Vitamin A supplementation
8. Vitamin K supplementation
9. Zinc supplementation
10. Selenium supplementation
11. Chlorhexidine treatment on the cord
12. Case management of neonatal sepsis and pneumonia
13. Kangaroo mother care (KMC)
14. Early breastfeeding
15. Thermal care
16. Application of continued distending pressure or CPAP to the lungs for RDS
17. Intravenous immune globulin (IVIG)
18. Surfactant therapy for RDS
19. Emollient therapy
20. Hand washing
21. Prophylaxis of eye infection
22. Use of Appropriate Low-cost Technology (incubators, techniques for minimally invasive intravenous access, protection against the excessive use of oxygen)

*Search term combinations*

**PubMed search terms**

(((((Infant[tiab] OR "Newborn"[tiab] OR "newborns"[tiab] OR "infants"[tiab] OR baby[tiab] OR babies[tiab] OR birth[tiab] OR neonates[tiab] OR neonate[tiab]) AND ("very low birth weight"[tiab] OR "VLBW"[tiab] OR "extremely low birth weight"[tiab] OR "ELBW"[tiab] OR "very low birthweight"[tiab] OR "extremely low birthweight"[tiab] OR "Very-low-birth-weight"[tiab] OR "very low birth-weight"[tiab] OR "extremely-low-birth-weight"[tiab] OR "extremely low birth-weight"[tiab] OR "preterm"[tiab] OR "pre-term"[tiab] OR "Premature"[tiab] OR "prematurity"[tiab])) OR ((("premature birth"[MeSH Terms]) OR "infant, low birth weight"[MeSH Terms]) OR "infant, premature"[MeSH Terms]) OR "infant, premature, diseases"[MeSH Terms])) AND ("Review"[Publication Type] OR "meta-analysis"[tiab] OR "metaanalysis"[tiab] OR "meta analysis"[tiab] OR "Search strategy"[All Fields] OR (("search"[tiab] AND ("literature"[tiab] OR "database"[tiab] OR "bibliographic"[tiab] OR "comprehensive"[tiab] OR "extensive"[tiab] OR "exhaustive"[tiab] OR "purposive"[tiab] OR "representative"[tiab] OR "synthesis"[tiab] OR "synthesise"[tiab] OR "systematic"[tiab])) OR (("Review"[tiab] AND (systematic[tiab] OR evidence[tiab] OR "Meta regression"[tiab] OR "meta synthesis"[tiab] OR "meta- synthesis"[tiab] OR "meta analysis"[tiab] OR metaanalysis[tiab] OR meta-analysis[tiab] OR literature[tiab] OR metanalysis[tiab] OR Metaregression[tiab] OR "Meta-regression"[tiab] OR "Methodological overview"[tiab] OR "Methodologically overview"[tiab] OR "pooled analysis"[tiab] OR "pooled data"[tiab] OR "Quantitative overview"[tiab] OR effectiveness[tiab] OR effects[tiab] OR synthesis[tiab] OR integrated[tiab] OR mapping[tiab] OR methodologial[tiab] OR methodologially[tiab] OR intervention[tiab] OR quantitative[tiab] OR research integration[tiab])) OR (("systematic"[tiab] AND ("overview"[tiab] OR "synthesis"[tiab])) OR "review literature as topic"[MeSH Terms]))))) NOT ("animals"[MeSH Terms] NOT ("humans"[MeSH Terms] AND "animals"[MeSH Terms]))

**British Nursing Index (BNI)**

**Subject Heading** - **Neonates : Birthweight**

[Used for] Low Birth Weight,

Neonates Premature,

Premature Babies,

Preterm Babies
1. (very low birth weight or VLBW or extremely low birth weight or ELBW or very low birthweight or extremely low birthweight or Very-low-birth-weight or very low birth-weight or extremely-low-birth-weight or extremely low birth-weight or Preterm or pre-term or Premature or Prematurity).mp. [mp=title, abstract, heading words]

2. (Infant or Newborn or Newborns or Infants or Baby or Babies or Birth or neonates or Neonate).mp. [mp=title, abstract, heading words]

3. Neonates : Birthweight/

4. 1 and 2

5. 3 or 4

6. (meta-analysis or metaanalysis or meta analysis or Search strategy).mp. [mp=title, abstract, heading words]

7. (search and (literature or database or bibliographic or comprehensive or extensive or exhaustive or purposive or representative or synthesis or synthesise or systematic)).mp. [mp=title, abstract, heading words]

8. (Review and (systematic or evidence or Meta regression or meta synthesis or meta-synthesis or meta analysis or metaanalysis or meta-analysis or literature or metanalysis or Metaregression or Meta-regression or Methodological overview or Methodologically overview or pooled analysis or pooled data or Quantitative overview or effectiveness or effects or synthesis or integrated or mapping or methodologial or methodologially or intervention or quantitative or research integration)).mp. [mp=title, abstract, heading words]

9. (systematic and (overview or synthesis)).mp. [mp=title, abstract, heading words]

10. 6 or 7 or 8 or 9

11. 5 and 10


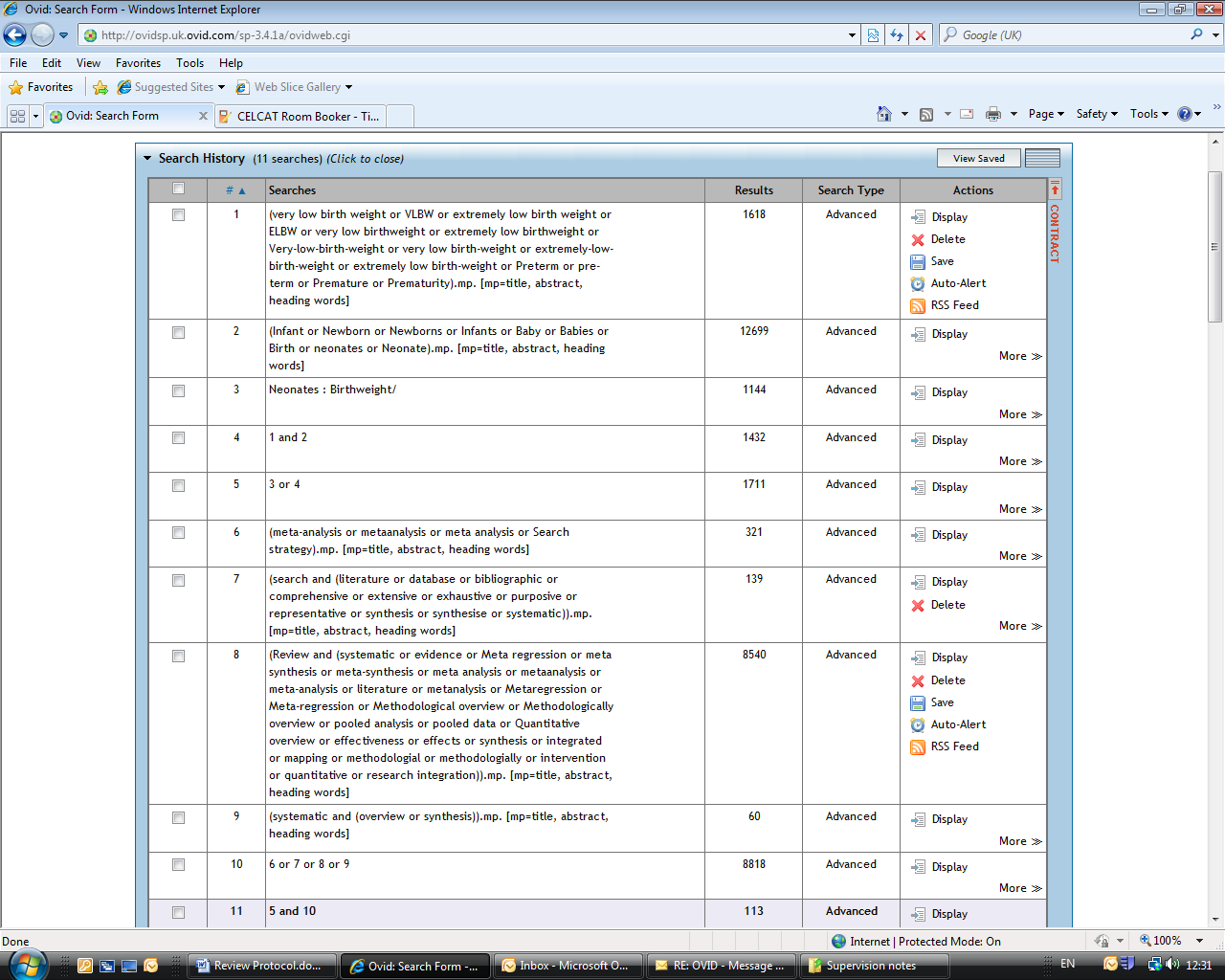


Figure 1 Search results from British Nursing Index

**EMBASE**

Subject Heading -

**systematic review**

[Used For]

review, systematic

**premature labor**

[Used For]

labor, premature

obstetric labor, premature

premature delivery

premature labour

preterm birth

preterm delivery

preterm labor

1. (((very low birth weight or VLBW or extremely low birth weight or ELBW or very low birthweight or extremely low birthweight or Very-low-birth-weight or very low birth-weight or extremely-low-birth-weight or extremely low birth-weight or Preterm or pre-term or Premature or Prematurity) and (Infant or Newborn or Newborns or Infants or Baby or Babies or Birth or neonates or Neonate)) or premature labor).ot,hw,ab,kw.

2. (meta-analysis or metaanalysis or meta analysis or Search strategy or (search and (literature or database or bibliographic or comprehensive or extensive or exhaustive or purposive or representative or synthesis or synthesise or systematic)) or (systematic and (overview or synthesis)) or (Review and (systematic or evidence or Meta regression or meta synthesis or meta-synthesis or meta analysis or metaanalysis or meta-analysis or literature or metanalysis or Metaregression or Meta-regression or Methodological overview or Methodologically overview or pooled analysis or pooled data or Quantitative overview or effectiveness or effects or synthesis or integrated or mapping or methodologial or methodologially or intervention or quantitative or research integration))).ot,hw,ab,kw.

3. "systematic review"/

4. 1 and (2 or 3)

5. limit 4 to human

**Health Management Information Consortium (HMIC)**

Controlled terms - premature babies/ or premature births/

1. premature babies/ or premature births/
2. ((very low birth weight or VLBW or extremely low birth weight or ELBW or very low birthweight or extremely low birthweight or Very-low-birth-weight or very low birth-weight or extremely-low-birth-weight or extremely low birth-weight or Preterm or pre-term or Premature or Prematurity) and (Infant or Newborn or Newborns or Infants or Baby or Babies or Birth or neonates or Neonate)).ab,hw,ti.
3. (1 or 2) and (systematic reviews or meta analysis or (meta-analysis or metaanalysis or meta analysis or Search strategy or (search and (literature or database or bibliographic or comprehensive or extensive or exhaustive or purposive or representative or synthesis or synthesise or systematic)) or (systematic and (overview or synthesis)) or (Review and (systematic or evidence or Meta regression or meta synthesis or meta-synthesis or meta analysis or metaanalysis or meta-analysis or literature or metanalysis or Metaregression or Meta-regression or Methodological overview or Methodologically overview or pooled analysis or pooled data or Quantitative overview or effectiveness or effects or synthesis or integrated or mapping or methodologial or methodologially or intervention or quantitative or research integration)))).ab,hw,ti.

37 results

**MEDLINE**

1. ((very low birth weight or VLBW or extremely low birth weight or ELBW or very low birthweight or extremely low birthweight or Very-low-birth-weight or very low birth-weight or extremely-low-birth-weight or extremely low birth-weight or Preterm or pre-term or Premature or Prematurity) and (Infant or Newborn or Newborns or Infants or Baby or Babies or Birth or neonates or Neonate)).ab,hw,ti.

2. (meta-analysis or metaanalysis or meta analysis or Search strategy or (search and (literature or database or bibliographic or comprehensive or extensive or exhaustive or purposive or representative or synthesis or synthesise or systematic)) or (systematic and (overview or synthesis)) or (Review and (systematic or evidence or Meta regression or meta synthesis or meta-synthesis or meta analysis or metaanalysis or meta-analysis or literature or metanalysis or Metaregression or Meta-regression or Methodological overview or Methodologically overview or pooled analysis or pooled data or Quantitative overview or effectiveness or effects or synthesis or integrated or mapping or methodologial or methodologially or intervention or quantitative or research integration))).ab,hw,ti.

3. Premature Birth/

4. "Review Literature as Topic"/

5. (1 or 3) and (2 or 4)

6. limit 5 to humans

5706 results

**PyscINFO**

1,806 to May Week 2 2011

1. ((very low birth weight or VLBW or extremely low birth weight or ELBW or very low birthweight or extremely low birthweight or Very-low-birth-weight or very low birth-weight or extremely-low-birth-weight or extremely low birth-weight or Preterm or pre-term or Premature or Prematurity) and (Infant or Newborn or Newborns or Infants or Baby or Babies or Birth or neonates or Neonate)).ab,hw,ti.

2. (meta-analysis or metaanalysis or meta analysis or Search strategy or (search and (literature or database or bibliographic or comprehensive or extensive or exhaustive or purposive or representative or synthesis or synthesise or systematic)) or (systematic and (overview or synthesis)) or (Review and (systematic or evidence or Meta regression or meta synthesis or meta-synthesis or meta analysis or metaanalysis or meta-analysis or literature or metanalysis or Metaregression or Meta-regression or Methodological overview or Methodologically overview or pooled analysis or pooled data or Quantitative overview or effectiveness or effects or synthesis or integrated or mapping or methodologial or methodologially or intervention or quantitative or research integration))).ab,hw,ti.

3. premature birth/

4. "literature review"/ or meta analysis/

5. (1 or 3) and (2 or 4)

6. limit 5 to human

**Social Policy and Practice (SPP)**

1. ((very low birth weight or VLBW or extremely low birth weight or ELBW or very low birthweight or extremely low birthweight or Very-low-birth-weight or very low birth-weight or extremely-low-birth-weight or extremely low birth-weight or Preterm or pre-term or Premature or Prematurity) and (Infant or Newborn or Newborns or Infants or Baby or Babies or Birth or neonates or Neonate)).ab,hw,ti.

2. (meta-analysis or metaanalysis or meta analysis or Search strategy or (search and (literature or database or bibliographic or comprehensive or extensive or exhaustive or purposive or representative or synthesis or synthesise or systematic)) or (systematic and (overview or synthesis)) or (Review and (systematic or evidence or Meta regression or meta synthesis or meta-synthesis or meta analysis or metaanalysis or meta-analysis or literature or metanalysis or Metaregression or Meta-regression or Methodological overview or Methodologically overview or pooled analysis or pooled data or Quantitative overview or effectiveness or effects or synthesis or integrated or mapping or methodologial or methodologially or intervention or quantitative or research integration))).ab,hw,ti.

3. 1 and 2

18 results from 3

3 results were selected

**Cochrane Library**

#1 ((very low birth weight or VLBW or extremely low birth weight or ELBW or very low birthweight or extremely low birthweight or Very-low-birth-weight or very low birth-weight or extremely-low-birth-weight or extremely low birth-weight or Preterm or pre-term or Premature or Prematurity) and (Infant or Newborn or Newborns or Infants or Baby or Babies or Birth or neonates or Neonate)):ti,ab,kw

#2 MeSH descriptor Infant, Premature, Diseases explode all trees 1943

#3 MeSH descriptor Infant, Premature explode all trees 2345

#4 MeSH descriptor Premature Birth explode all trees 211

#5 MeSH descriptor Infant, Low Birth Weight explode all trees 1586

#6 (#1 OR #2 OR #3 OR #4 OR #5) 7511

#7 (meta-analysis or metaanalysis or meta analysis or Search strategy or (search and (literature or database or bibliographic or comprehensive or extensive or exhaustive or purposive or representative or synthesis or synthesise or systematic)) or (systematic and (overview or synthesis)) or (Review and (systematic or evidence or Meta regression or meta synthesis or meta-synthesis or meta analysis or metaanalysis or meta-analysis or literature or metanalysis or Metaregression or Meta-regression or Methodological overview or Methodologically overview or pooled analysis or pooled data or Quantitative overview or effectiveness or effects or synthesis or integrated or mapping or methodologial or methodologially or intervention or quantitative or research integration))):ti,ab,kw 31635

#8 MeSH descriptor Review Literature as Topic explode all trees 96

#9 (#6 AND ( #7 OR #8 )) 664

**DARE/HTA**

1 (((very low birth weight or VLBW or extremely low birth weight or ELBW or very low birthweight or extremely low birthweight or Very-low-birth-weight or very low birth-weight or extremely-low-birth-weight or extremely low birth-weight or Preterm or pre-term or Premature or Prematurity) and (Infant or Newborn or Newborns or Infants or Baby or Babies or Birth or neonates or Neonate))) 963

2 MeSH DESCRIPTOR Premature Birth EXPLODE ALL TREES 53

3 MeSH DESCRIPTOR Infant, Low Birth Weight EXPLODE ALL TREES 94

4 MeSH DESCRIPTOR Infant, Premature, Diseases EXPLODE ALL TREES 95

5 #1 OR #2 OR #3 OR #4 979

6 MeSH DESCRIPTOR Infant, Premature EXPLODE ALL TREES 139

7 #5 OR #6 979

8 MeSH DESCRIPTOR Review Literature as Topic EXPLODE ALL TREES 52

9 (meta-analysis or metaanalysis or meta analysis or Search strategy or (search and (literature or database or bibliographic or comprehensive or extensive or exhaustive or purposive or representative or synthesis or synthesise or systematic)) or (systematic and (overview or synthesis)) or (Review and (systematic or evidence or Meta regression or meta synthesis or meta-synthesis or meta analysis or metaanalysis or meta-analysis or literature or metanalysis or Metaregression or Meta-regression or Methodological overview or Methodologically overview or pooled analysis or pooled data or Quantitative overview or effectiveness or effects or synthesis or integrated or mapping or methodologial or methodologially or intervention or quantitative or research integration))) 29747

10 #7 AND (#8 OR #9) 840

**Cumulative Index to Nursing and Allied Health Literature (CINAHL)**


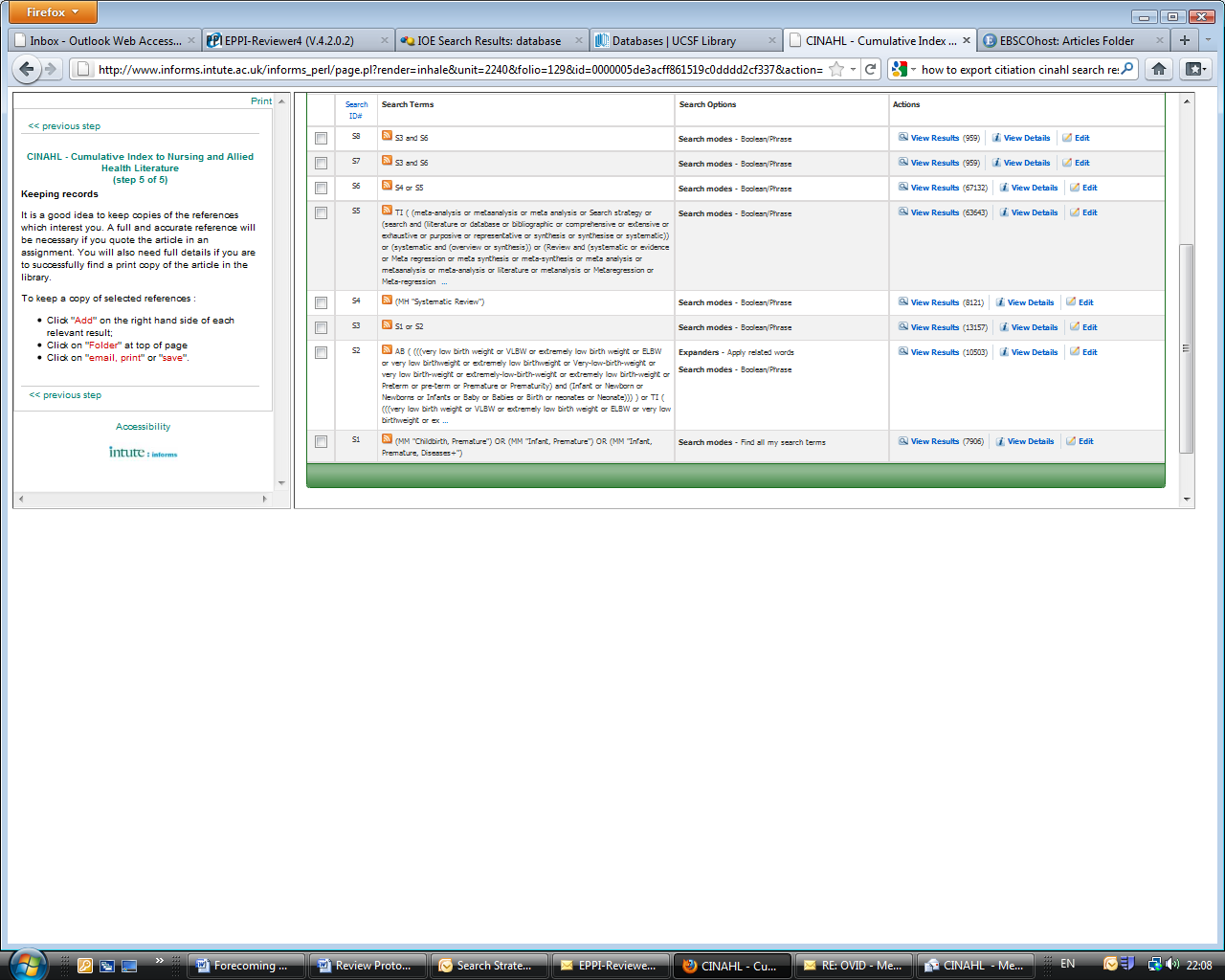


Figure 2 Search results from CINAHL

959 S7 S3 and S6 Search modes - Boolean/Phrase Interface - EBSCOhost
Search Screen - Advanced Search
Database - CINAHL 959 S6 S4 or S5 Search modes - Boolean/Phrase Interface - EBSCOhost
Search Screen - Advanced Search
Database - CINAHL 67132 S5 TI ( (meta-analysis or metaanalysis or meta analysis or Search strategy or (search and (literature or database or bibliographic or comprehensive or extensive or exhaustive or purposive or representative or synthesis or synthesise or systematic)) or (systematic and (overview or synthesis)) or (Review and (systematic or evidence or Meta regression or meta synthesis or meta-synthesis or meta analysis or metaanalysis or meta-analysis or literature or metanalysis or Metaregression or Meta-regression or Methodological overview or Methodologically overview or pooled analysis or pooled data or Quantitative overview or effectiveness or effects or synthesis or integrated or mapping or methodologial or methodologially or intervention or quantitative or research integration))) ) or AB ( (meta-analysis or metaanalysis or meta analysis or Search strategy or (search and (literature or database or bibliographic or comprehensive or extensive or exhaustive or purposive or representative or synthesis or synthesise or systematic)) or (systematic and (overview or synthesis)) or (Review and (systematic or evidence or Meta regression or meta synthesis or meta-synthesis or meta analysis or metaanalysis or meta-analysis or literature or metanalysis or Metaregression or Meta-regression or Methodological overview or Methodologically overview or pooled analysis or pooled data or Quantitative overview or effectiveness or effects or synthesis or integrated or mapping or methodologial or methodologially or intervention or quantitative or research integration))) ) Search modes - Boolean/Phrase Interface - EBSCOhost
Search Screen - Advanced Search
Database - CINAHL 63643 S4 (MH "Systematic Review") Search modes - Boolean/Phrase Interface - EBSCOhost
Search Screen - Advanced Search
Database - CINAHL 8121 S3 S1 or S2 Search modes - Boolean/Phrase Interface - EBSCOhost
Search Screen - Advanced Search
Database - CINAHL 13157 S2 AB ( (((very low birth weight or VLBW or extremely low birth weight or ELBW or very low birthweight or extremely low birthweight or Very-low-birth-weight or very low birth-weight or extremely-low-birth-weight or extremely low birth-weight or Preterm or pre-term or Premature or Prematurity) and (Infant or Newborn or Newborns or Infants or Baby or Babies or Birth or neonates or Neonate))) ) or TI ( (((very low birth weight or VLBW or extremely low birth weight or ELBW or very low birthweight or extremely low birthweight or Very-low-birth-weight or very low birth-weight or extremely-low-birth-weight or extremely low birth-weight or Preterm or pre-term or Premature or Prematurity) and (Infant or Newborn or Newborns or Infants or Baby or Babies or Birth or neonates or Neonate))) ) Expanders - Apply related words
Search modes - Boolean/Phrase Interface - EBSCOhost
Search Screen - Advanced Search
Database - CINAHL 10503 S1 (MM "Childbirth, Premature") OR (MM "Infant, Premature") OR (MM "Infant, Premature, Diseases+") Search modes - Find all my search terms Interface - EBSCOhost
Search Screen - Advanced Search
Database - CINAHL

**Turning Research Into Practice (TRIP)**


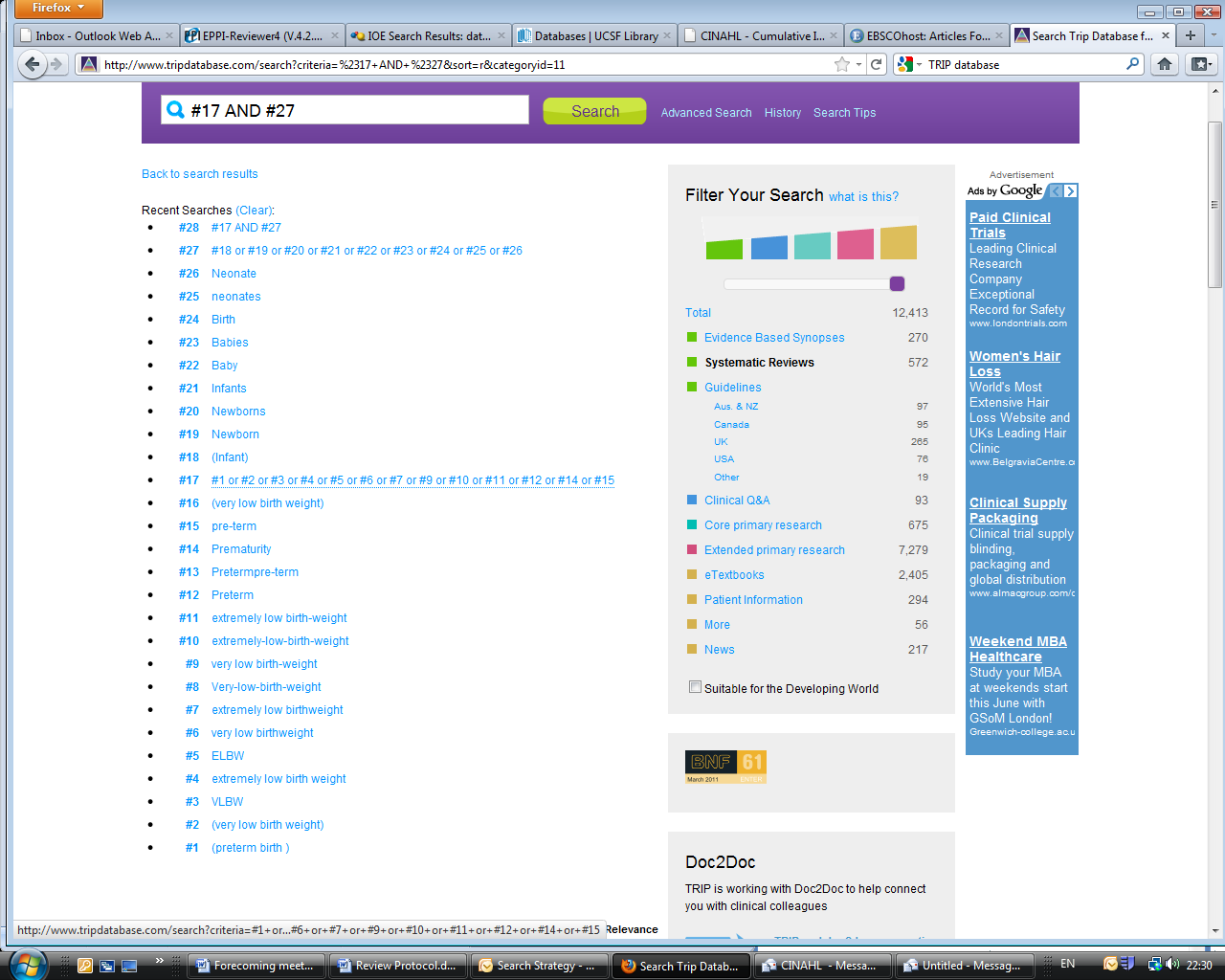
**Figure 3** Search results from TRIP

**POPLINE**

1. ((very low birth weight / VLBW / extremely low birth weight / ELBW / very low birthweight / extremely low birthweight / Very-low-birth-weight / very low birth-weight / extremely-low-birth-weight / extremely low birth-weight / Preterm / pre-term / Premature / Prematurity) and (Infant / Newborn / Newborns / Infants / Baby / Babies / Birth / neonates / Neonate))
2. (systematic reviews / meta analysis / (meta-analysis / metaanalysis / meta analysis / Search strategy / (search and (literature or database or bibliographic or comprehensive or extensive or exhaustive or purposive or representative or synthesis or synthesise or systematic)) or (systematic and (overview or synthesis)) or (Review and (systematic or evidence or Meta regression or meta synthesis or meta-synthesis or meta analysis or metaanalysis or meta-analysis or literature or metanalysis or Metaregression or Meta-regression or Methodological overview or Methodologically overview or pooled analysis or pooled data or Quantitative overview or effectiveness or effects or synthesis or integrated or mapping or methodologial or methodologially or intervention or quantitative or research integration)))).ab,hw,ti.
