# Supplementary material for: Top research priorities for preterm birth: results of a prioritisation partnership between people affected by preterm birth and healthcare professionals
Source: BMC Pregnancy Childbirth. 2019 Dec 30;19:528. doi: 10.1186/s12884-019-2654-3 (PMC6938013; doi:10.1186/s12884-019-2654-3)
Supplement: Supplementary file 3 — Additional file 3. Long list of questions sent for voting. [file 12884_2019_2654_MOESM3_ESM.docx]

**Additional file 3**

**Long list of 104 questions sent for voting, by category and source**

*Key:*

SV=uncertainty from **survey,** more than one person has suggested a similar uncertainty so these are grouped and the summary question reflects all similar questions

SR=uncertainty from **systematic reviews**

CG: uncertainty from **clinical guidance**

| **Category** | **Subcategory** | **Question** | **Source** |
| --- | --- | --- | --- |
| Pre-pregnancy or early pregnancy treatments | Treatments to predict or prevent preterm birth | Which treatments (including diagnostic tests) are most effective to predict or prevent first [preterm birth](#preterm)? | SV |
|  | Treatments to prevent preterm birth in next pregnancy | What treatments can predict reliably the likelihood of subsequent infants being [preterm](#preterm)? | SV |
|  |  | Is treating [fibroids](#fibroids) during pregnancy effective to reduce the risk of preterm birth? | SV |
|  |  | What treatments are effective to prevent [fibroids](#fibroids) growing during pregnancy and thus reduce the risk of preterm birth? | SV |
|  | Treatments to prevent [pre-eclampsia](#preeclampsia) | Which treatments are most effective to prevent [pre-eclampsia](#preeclampsia) (for example, [progesterone](#hormone), calcium, garlic etc)? | SR |
|  |  | When should women who have [pre-eclampsia](#preeclampsia) with mild or moderate [hypertension](#hypertension) give birth? | CG |
| Treatments during pregnancy ([antenatal](#antenatal)) | [Screening](#screening)/early diagnosis of preterm birth | Is [screening](#screening) in the first [trimester](#trimester) effective to help prevent preterm birth? | SV |
|  |  | Can [screening](#screening) of the [placenta](#placenta) be effective to detect [placenta](#placenta) abnormalities associated with [preterm birth](#preterm)? | SV |
|  |  | What methods are most effective to predict risk of preterm birth in order to allocate service provision? | SV |
|  |  | Is routine [transvaginal scanning](#transvaginal) during pregnancy to detect short [cervical length](#cerclage), and treatment, cost effective? | SV |
|  |  | Does specialist prenatal care, for women showing signs of [preterm birth](#preterm), improve [morbidity](#morbidity) and [mortality](#mortality) in mother and baby? | SV |
|  |  | Does [screening](#screening) and treatment for Group B Streptococcus help to prevent [preterm birth](#preterm) and neonatal [morbidity](#morbidity) and [mortality](#mortality)? | SV |
|  |  | Are [risk scoring systems](#scoring) effective to help predict [preterm birth](#preterm)? | SV |
|  |  | Which treatments are effective in preventing spontaneous preterm birth in women with twin and triplet pregnancies, especially in those at high risk of preterm birth? | CG |
|  |  | What is the [effectiveness](#effectiveness), practicality and acceptability of [chlamydia](#chlamydia) screening to prevent [preterm birth](#preterm) in an [antenatal](#antenatal) setting? | CG |
|  | Medications or substance intake during pregnancy (incl vitamins) | Are vitamins effective to prevent preterm birth? | SV |
|  |  | How do stress, trauma and physical workload contribute to the risk of [preterm birth](#preterm), are there effective ways to reduce those risks and does modifying those risks alter [outcome](#outcome)? | SV |
|  |  | What dosage of [progesterone](#hormone) is most effective for preventing [preterm birth](#preterm) in high risk groups? | SV, SR |
|  |  | Does [nutritional deficiency](#deficiency) influence the [effectiveness](#effectiveness) of treatments known to be effective for the prevention of preterm birth? | SV, SR |
|  | Information provision and service delivery during pregnancy | What guidance and information is most useful for parents at risk of having preterm infants? | SV |
|  |  | Does education and support, for mothers with increased risk of [preterm birth](#preterm) and their families, help to prevent preterm birth? | SV |
|  |  | Which test is most effective to diagnose [urinary tract infections](#urinary) in early labour and does early detection and treatment of [urinary tract infection](#urinary) affect preterm birth and its [outcomes](#outcome)? | SV, SR |
|  |  | How should healthcare professionals best communicate, including information about possible disabilities, with parents at risk of [preterm birth](#preterm) to improve outcomes? | SV |
|  | Length of cervix/cervical [cerclage](#cerclage) (stitching) | Does cervical [cerclage](#cerclage) help to prevent preterm birth? | SV, CG, SR |
|  |  | Does the use of pre-pregnancy diagnostic techniques aimed at diagnosing ‘[cervical weakness’](#weakness) in women with a history of preterm birth and/or second-[trimester](#trimester) loss assist in the decision to place a history-indicated [cerclage](#cerclage) and does this influence outcome? | SV |
|  |  | In women who have an incidental finding of a short [cervical length](#cerclage), does analysis of amniotic fluid guide who benefits from cervical [cerclage](#cerclage)? | SV, SR |
|  |  | Does [pessary (progesterone)](#progesterone) help to prevent preterm birth? | SV |
|  |  | Should the decision on how best to minimise the risk of recurrent preterm birth in women at risk, either because of poor history of a short or dilated cervix, be ’personalised’, based on the clinical circumstances, skill and expertise of the clinical team and, most importantly, the woman’s informed choice? | SV |
|  | Other [antenatal](#antenatal) Treatments | Is [tocolytic therapy](#tocolysis) effective to prevent preterm birth in multiple pregnancies? | SV, CG |
|  |  | In women threatening preterm labour is maintenance [tocolytic therapy](#tocolysis) effective in improving [outcomes](#outcome) of preterm birth? | SV, CG |
|  |  | Which lifestyle changes including gym, bed rest, posture and sexual intercourse are effective to minimise the risk of preterm birth? | SV, SR |
|  |  | What are the benefits and harms of [immediate versus deferred delivery](#deferred) of preterm infants with suspected fetal compromise? | SR |
| Treatments at birth | [Premature rupture of membranes (PROM)](#membranes) | Does intervening after a specific duration of [PROM](#membranes) affect incidence and [outcome](#outcome) of [preterm birth](#preterm)? | SV, |
|  |  | Which treatments are most effective for PROM | SV |
|  |  | What are the clinical [benefits and harms](#benefits) for women and their babies of immediate delivery compared with [expectant management](#expectant) for women with preterm pre-labour rupture of the membranes? | SR |
|  |  | What are the [risks and benefits](#benefits) of [expectant management](#expectant) versus delivery in women whose membranes rupture spontaneously between 34 and 37 weeks [gestation](#gestation)? | SV |
|  | [Caesarean](#caesarean) Section | Is birth by [caesarean](#caesarean) section of benefit to neonatal [outcome](#outcome) for late preterm infants? | SV |
|  | Prophylactic corticosteroid therapy in preterm labour (steroids given before/during or after birth) | What are the long-term [benefits and risks](#benefits) of multiple courses of [antenatal](#antenatal) [corticosteroids](#corticosteroids) on neonatal [morbidity](#morbidity) (including early childhood) and [mortality](#mortality)? | CG |
|  | Delayed cord clamping | What is the best time to clamp the [umbilical cord](#umbilical) for preterm babies? | SV, SR |
|  | Other perinatal treatments | Is [antenatal](#antenatal) and [intrapartum](#intrapartum) [cardiotocography](#cardiotocogratography) monitoring of preterm infants beneficial in improving [outcome](#outcome)s of preterm birth? | SV, |
|  |  | What roles should fathers be offered during preterm labour and preterm birth? | SV |
|  |  | What are the benefits of specialist [antenatal](#antenatal) care at preterm birth clinics for women at high risk of preterm birth? | SV |
| Treatments after birth (postnatal) until discharge from hospital | Initial care and support at birth | Should extremely immature babies be [intubated](#intubated) at delivery and [surfactant](#surfactant) given, or should [CPAP](#CPAP) be attempted? | SV |
|  |  | What should be included in the care offered to babies during the initial stabilisation after preterm birth? | SV |
|  |  | What is the best treatment for life-threatening lung damage in preterm infants? | SV |
|  |  | Which [interventions](#intervention) should be included in a package of respiratory care to optimise developmental outcomes for preterm babies? | SV |
|  |  | Is h[igh flow nasal cannula (HFNC)](#HFNC) safe and effective as a form of respiratory support in preterm infants? | SR |
|  | Care and feeding during hospital | What is the best way to encourage [Kangaroo Mother Care](#Kangaoo) more by staff in [NICU](#NICU) or parents? | SV |
|  |  | Is community initiation of [Kangaroo Mother Care](#Kangaoo) beneficial? | SR |
|  |  | How effective is the use of early onset continuous [Kangaroo Mother Care](#Kangaoo) in stabilized preterm infants as an alternative to conventional neonatal care in resource limited settings? | SR |
|  |  | What type of support is most effective at improving breastfeeding in [NICU](#NICU)/SCBU/feeding clinics? | SV |
|  |  | What is the impact of length of [orogastric / nasogastric feeding](#orogastric) and reflux on early feeding development in preterm infants? | SV |
|  |  | Does [ad libitum](#ad) or demand/semi-demand feeding regimen (versus scheduled interval feeding) affect important [clinical outcomes](#outcome) for preterm infants and their families? | SV |
|  |  | What is the optimum milk feeding regimen, for preterm infants, including quantity and speed of feeding and use of donor and formula milks? | SV, SR |
|  |  | How does [banked preterm milk](#milk) versus banked term milk compare to promote growth and development in very low birth weight infants? | SR |
|  |  | At what time is it safest to introduce formula milk to preterm infants when expressed breast milk is unavailable? | SV |
|  |  | Does slow advancement of [enteral feed volumes](#enterel) help to prevent [necrotising enterocolitis](#NEC) in very low birth weight infants? | SV |
|  |  | What is the best method to diagnose feeding problems in preterm infants, including allergies and failure to thrive due to poor feeding? | SV |
|  |  | What are the effects of [oral motor](#oro) treatments versus treatments such as managing flow rate, pacing during [oral feeds](#oral) and [thickening](#thickening) of feedings, on pulmonary function in preterm infants? | SV, SR |
|  |  | Which treatments should be included in a care bundle to optimise nutrition, growth and [oro–motor](#oro) development in premature babies with feeding difficulties? | SR |
|  |  | Is breast milk fortifier beneficial for preterm infants? | SV |
|  |  | How is it best to establish [oral feeding](#oral) in preterm infants? | SV |
|  |  | Which treatments are most effective for [necrotising enterocolitis](#NEC) in preterm infants? | SV |
|  |  | Can [neurodevelopmental](#developmental) care and *strong/effective* parenting improve quality of life for preterm infants? | SV |
|  |  | Is the newborn individualised development care and assessment program (NIDCAP) effective to improve the quality of care in preterm infants? | SV |
|  |  | Is routine use of [ethamsylate](#ethamsylate) effective to improve neurodevelopment and [mortality](#mortality) in preterm infants? | SV, SR |
|  |  | Which treatments are most effective to prevent necrotising enterocolitis in preterm infants? | SR |
|  | [Bonding](#bonding) and [attachment](#attachment) of parents and infants | Which treatments improve [attachment](#attachment) and [bonding](#bonding) and does the promotion of appropriate [attachment](#attachment) and [bonding](#bonding) improve outcomes? | SV |
|  | Effective communication/support to parents | What emotional and practical support should be included in a care bundle that aims to optimise outcomes of preterm birth? | SV |
|  | Sensory issues | What are the best ways to optimise the environment in order to improve outcomes (for example [cycled light](#light), eye masks, ear muffs or [music](#music) therapy)? | SV, SR |
|  | Pain management of infants | How should [morphine](#morphine) or ketamine for ventilated preterm babies be used to optimise outcomes? | SV |
|  |  | How should [sucrose](#sucrose) be used to optimise outcomes? | SR |
|  |  | Are pharmacological treatments, including [analgesia](#analgesia), effective for pain management in preterm infants? | SV, SR |
|  |  | Which [non-pharmacological](#Non) measures relieve pain in the vulnerable group of sick and ventilated preterms? | SR |
|  |  | What is the best way to judge whether a baby is feeling pain (for example, by their face, behaviours or brain activities)? | SV, SR |
|  | Other postnatal treatments during hospital stay | Do preterm babies have better outcomes if their parents have roomed in? | SV |
|  |  | What is role of siblings in caring for their preterm sibling in hospital? | SV |
|  |  | Do parents of preterm infants benefit from an open approach to notes and ward rounds? | SV |
|  |  | What is the benefit of diagnostic testing, including [lumbar puncture](#lumbar), for preterm infants? | SV |
|  |  | How should neonatal transport of preterm babies be done to minimise brain injury? | SV |
|  |  | Does [stem cell therapy](#stem) improve outcomes in preterm babies with evidence of brain injury? | SV |
|  |  | What is the role of therapeutic [hypothermia](#hypothermis) in premature babies? | SV |
|  |  | How can infection in preterm infants be better prevented? | SV |
|  |  | What is the best way to manage early-onset neonatal infection for preterm babies? | SV |
|  |  | What is the clinical and cost [effectiveness](#effectiveness) of [intrapartum](#intrapartum) prophylactic antiobiotic [benzylpenicillin](#benzylpenicillin) to prevent early onset neonatal infection? | CG |
|  |  | What are the best methods, including laboratory investigations, to identify risk factors and clinical signs and symptoms to identify babies needing antibiotics for early-onset neonatal infection? | CG |
|  |  | What is the clinical and cost [effectiveness](#effectiveness) of laboratory investigations used individually or in combination to exclude early-onset neonatal infection in babies receiving antibiotics for suspected infection? | CG |
|  |  | What is the optimal duration of treatment in infants receiving antibiotics for confirmed early onset neonatal infections? | CG |
|  |  | How does each step in the care pathway for prevention and treatment of early-onset neonatal infection impact on babies and their families? | CG |
|  |  | What is the clinical and cost-[effectiveness](#effectiveness) of information and support offered to parents and carers of babies who have received antibiotics for suspected or proven early-onset neonatal infection? | CG |
|  |  | Which risk factors for early-onset neonatal infection, symptoms and signs of infection, and laboratory investigations should be used to identify babies who should receive antibiotics? | CG |
|  |  | Is drainage, irrigation and fibrinolytic therapy ([DRIFT](#DRIFT)) an effective treatment in the management of post-haemorrhagic hydrocephalus in preterm infants who may suffer severe disability as a result? | CG |
|  |  | What should be included in the care of jaundice in premature babies to optimize outcomes? | CG |
| Treatments after birth (postnatal) after discharge from hospital | End of life care | What should be included in packages of care to support parents (and families) with decisions about continuing or withdrawing life-sustaining care? | SV |
|  |  | What should be included in packages of care to support parents (and families) when a premature baby dies? | SV |
|  | During transition from hospital to home | What should be included in packages of care to support parents and families / carers when a premature baby is discharged from hospital? | SV |
|  |  | Is multi-nutrient fortified breast milk compared with unfortified breast milk more effective for long term growth and development of preterm infants following hospital discharge? | SV |
|  |  | Which nutrients are most effective for growth and development of preterm infants? | SV |
| General / Patient Care | | Is the UK neonatal care model compared to the [Swedish care model](#swedish) more effective for the care of [preterm infants](#preterm)? | SV |
|  | | What is the effect of partnership working between healthcare professionals and parents on the care of [preterm infants](#preterm)? | SV |
| Staff Issues | | What should healthcare professionals know about [preterm birth](#preterm) (staff training about [preterm birth](#preterm))? | SV |
|  | | What level of training about preterm birth is optimal for midwives? | SV |
|  | | Do support groups for healthcare professionals involved in the care of [preterm babies](#preterm) (and women at risk of [preterm birth](#preterm)) improve outcomes for the babies (or the staff)? | SV |
|  | | What should the role of the specialist neonatal nurse be in the team in order to achieve the best long-term [developmental](#developmental) outcomes for infants and their families in neonatal care? | SV |
