# Supplementary material for: Top research priorities for preterm birth: results of a prioritisation partnership between people affected by preterm birth and healthcare professionals
Source: BMC Pregnancy Childbirth. 2019 Dec 30;19:528. doi: 10.1186/s12884-019-2654-3 (PMC6938013; doi:10.1186/s12884-019-2654-3)
Supplement: Supplementary file 4 — Additional file 4. Organisations invited to participate. [file 12884_2019_2654_MOESM4_ESM.pdf]

Organisations contacted and invited to participate in the priority setting partnership

1. "Association of Early Pregnacy Units
2. The Association of Paediatric Anaesthetists of Great Britain and Ireland
3. "Action on Pre-eclampsia"
4. Association of Paediatric Emergency Medicine
5. "Antenatal Results and Choices"
6. British Academy of Childhood Disability
7. British Association of General Paediatrics
8. "British Association of Perinatal Medicine"
9. British Congenital Cardiac Association
10. "Bliss, the special care baby charity"
11. "The British Maternal and Fetal Medicine Society "
12. British Paediatric Allergy, Immunity & Infection Group
13. British Paediatric Neurology Association
14. British Paediatric Respiratory Society
15. British Society of Paediatric Radiology
16. The Cleft Lip and Palate Association
17. James Lind Alliance
18. "The Multiple Births Foundation"
19. MCRN Neonatal Clinical Studies Advisory Group
20. the Neonatal Nurses Association
21. Obsteteric Anaesthetists Association
22. "The Royal College of Midwives"
23. "The Royal College of Nursing"
24. Royal College of Anaesthetists

25. The Royal College of Paediatrics and Child Health
26. The Royal College of Surgeons
27. "The Royal College of Obstetricians and Gynaecologists
28. "Stillbirth and Neonatal Death Society"
29. "Scope -about cerebral palsy. For disabled people achieving equality"
30. "TAMBA - The Twin and Multiple Birth Association "
31. TinyLife - the premature baby charity for N.I.
32. "Tommy's [Let's talk baby]"
33. "the UK Twin to Twin Transfusion Syndrome Association"
34. British Paediatric Pathology Group
35. "Cochrane Neonatal Group"
36. "Cochrane Pregnancy and Childbirth Group"
37. Irish PrematureBabies
38. Miscarriage Association
39. "NHS Evidence Women's Health"
40. The British Association of Paediatric Surgeons
41. The Children's Trust
42. The Paediatric Intensive Care Society
