# Supplementary material for: Top research priorities for preterm birth: results of a prioritisation partnership between people affected by preterm birth and healthcare professionals
Source: BMC Pregnancy Childbirth. 2019 Dec 30;19:528. doi: 10.1186/s12884-019-2654-3 (PMC6938013; doi:10.1186/s12884-019-2654-3)
Supplement: Supplementary file 5 — Additional file 5. Submissions formatted as research questions. [file 12884_2019_2654_MOESM5_ESM.pdf]

# Uncertainties and how they were formatted into research questions

Submissions were formatted into 'PICO' questions, with participants, intervention, comparator, and outcomes

*Key:*

SV=uncertainty from **survey,** more than one person has suggested a similar uncertainty so these are grouped and the summary question reflects all similar questions

SR=uncertainty from **systematic reviews**

CG: uncertainty from **clinical guidance**

## 1.1 PRE-PREGNANCY INTERVENTIONS

### 1.1.1 INTERVENTIONS TO PREDICT OR PREVENT PRETERM BIRTH

| Source | Uncertainties | Reference | Notes |
|---|---|---|---|
| **SV** | Are there new ways to predict or prevent preterm birth or preterm labour?<br>Which interventions are most effective to predict or prevent preterm birth? | Service user (23), HCP (8), both (2) | |
| **SV** | Which interventions can prevent necrotising enterocolitis (NEC)?<br>Which interventions are most effective to prevent necrotising enterocolitis in preterm infants? | Both (1), Service user (2), HCP (1), | |

### 1.1.2 INTERVENTIONS TO PREVENT SUBSEQUENT PREGNANCY

| Source | Uncertainties | Reference | Notes |
|---|---|---|---|
| **SV** | What is the likelihood for subsequent children being born preterm?<br>What is the likelihood of subsequent infants being preterm? | Service user (6), both (1) | |
| **CG** | What are the risks of preterm birth after previous caesarean birth?<br><br>What is the risk of preterm birth after a previous caesarean section? | Green-top Guideline No. 45 | |

### 1.1.3 INTERVENTIONS TO PREVENT PRE-ECLAMPSIA

| Source | Uncertainties | Reference | Notes |
|---|---|---|---|
| **SV** | Which interventions can prevent pre-eclampsia?<br>Which interventions are most effective to prevent pre-eclampsia? | Service user (24), Both (3) | |
| **SR** | Can progesterone prevent pre-eclampsia and its complications?<br>Is progesterone effective to help prevent pre-eclampsia and its complications? | Progesterone for preventing pre-eclampsia and its complications | |
| **CG** | When should women who have pre-eclampsia with mild or moderate hypertension give birth? | NICE clinical guideline 107 | |

## 1.2 INTERVENTIONS DURING PREGNANCY (ANTENATAL)

### 1.2.1 SCREENING/EARLY DIAGNOSIS OF PRETERM BIRTH

| Source | Uncertainties | Reference | Notes |
|---|---|---|---|
| **SV** | Is screening in the first trimester effective to help prevent preterm birth? | No input (1),<br>HCP (1) | |
| **SV** | Can/how we screen abnormality of placenta? Is it possible, and how, can we screen for placenta abnormalities?<br>Is screening of the placenta effective to detect placenta abnormalities? | Service User (2) | |
| **SV** | What methods are most effective to predict risk of preterm birth in order to allocate service provision? | Both(1), HCP (2), Service User (2)<br>No input (1) | |
| **SV** | Is routine transvaginal scanning during pregnancy to detect short cervical length, and treatment, cost effective? | HCP(1)<br>Service user (1) | |
| **SV** | Does specialist prenatal care, for women showing signs of preterm birth, improve morbidity and mortality in mother and baby? | Service user(1)<br>Both (1) | |
| **SV** | Does screening and treatment for Group B Stretococcus help to prevent preterm birth and neonatal morbidity and mortality? | Service user(3) | |
| **SV** | How screening tools including checking cervical length, other than vaginal swab, are effective? How effective are additional screening tools, including checking cervical length, other than vaginal swab?<br>What screening tools, other than vaginal swab, are effective to measure cervical length? | HCP (1) | |
| **SV** | What is the best method to diagnose short cervical length? What are the indications for short cervical length? Is routine cervical length measurement effective to predict preterm birth? | Service User (1), both (1) | |
| **SR** | Do risk scoring systems help to predict preterm birth?<br>Are risk scoring systems effective to help predict preterm birth? | Davey (2010) Risk scoring systems for predicting preterm birth with the aim of reducing associated adverse outcomes | |
| **CG** | Which interventions are effective in preventing spontaneous preterm birth in women with twin and triplet pregnancies, especially in those at high risk of preterm birth? | NICE clinical guideline 129 | |
| **CG** | What is the effectiveness, practicality and acceptability of chlamydia screening in an antenatal setting? | NICE clinical guideline 62 Antenatal care | |

## 1.2.2 MEDICATIONS OR SUBSTANCE INTAKE DURING PREGNANCY (INCL VITAMINS)

| Source | Uncertainties | Reference | Notes |
|---|---|---|---|
| **SV** | Are vitamins effective to prevent preterm birth? | Service user(1) | |
| **SV** | What are risk factors of preterm birth in relation to stress, trauma, or physical workload? How do stress, trauma or physical workload contribute to the risk of preterm birth?<br>How do stress, trauma and physical workload contribute to the risk of preterm birth? | HCP(1), Service user(1) | |
| **SV, SR** | Is it worth considering the use of progesterone therapy for preventing preterm birth in individual high-risk women, if so, which dose of progesterone prevents preterm birth?<br><br>What dosage of progesterone is most effective for preventing preterm birth in high risk groups? | Service user(2)<br>Both (1), Smith et al (2009) | |
| **SR** | For future research efforts, the best strategy would be to continue to test promising interventions in populations with and without nutritional deficiency in large multicenter trials that require short time periods (168). However, it should be put in scientific context that for most of pregnancy-specific conditions such as preeclampsia, postpartum hemorrhage (which, when defined as losses .1000 mL, still has an incidence of 3–5% of deliveries), preterm delivery, premature rupture of the membranes and initiation of labor, we have limited knowledge of their etiology. Therapeutic options for these conditions remain limited to pregnancy termination or palliative treatments to be started when the condition is already manifest.<br>Does nutritional deficiency influence the effectiveness of interventions known to be effective for the prevention of preterm birth? | Villar José, Merialdi Mario, Gülmezoglu A Metin M; Abalos Edgardo, Carroli Guillermo, Kulier Regina, de Onis Mercedes (2003) Nutritional interventions during pregnancy for the prevention or treatment of maternal morbidity and preterm delivery: an overview of randomized controlled trials.. The Journal of nutrition. 133(5 Suppl 2): 1606S-1625S. | |

## 1.2.3 INFORMATION PROVISION AND SERVICE DELIVERY DURING PREGNANCY

| Source | Uncertainties | Reference | Notes |
|---|---|---|---|
| **SV** | What guidance and information can be provided to parents about preterm birth?<br>What guidance and information is most useful for parents at risk of having preterm infants? | Service user (1), both (2) | |
| **SV** | Does education and support, for mothers with increased risk of preterm birth and their families, help to prevent preterm birth? | HCP (2)<br>Service User (6)<br>Both (1), | |
| **SV, SR** | What is the best test to distinguish UTI at early stages of labour?<br>Which is the best class, route or regimen of antibiotic to treat symptomatic UTIs during pregnancy?<br><br>Which test is most effective to diagnose urinary tract in early labour?<br>Which antibiotic, method of administration and regimen is most effective to treat symptomatic urinary tract infection in pregnancy? | Both (1), HCP (1)<br>Treatments for symptomatic urinary tract infections during pregnancy | |
| **SV** | How should health care professionals communicate with service users to improve outcomes of preterm birth? For example, consequences of preterm birth such as explanation about babies' possible disabilities.<br>How should healthcare professionals communicate, including information about possible disabilities, with parents at risk of preterm birth to improve outcomes? | Service User (11), both (4), hcp(1) | |

## 1.2.4 LENGTH OF CERVIX/CERVICAL CERCLAGE (STITCHING)

| Source | Uncertainties | Reference | Notes |
|---|---|---|---|

| | | | |
|---|---|---|---|
| **SV, CG, SR** | Does cervical cerclage help to prevent preterm birth? | Service User (1) HCP (2), Both (1), Alfirevic (2011), Placenta praevia, placenta praevia acCGeta and vasa praevia: diagnosis and management - Green-top Guideline No. 27 | |
| **SV, SR** | Does cervical cerclage help to prevent preterm birth in women with a short uterine anomaly? | Service user (1), Both (1), Alfirevic (2011) | |
| **CG** | Does the use of pre-pregnancy diagnostic techniques aimed at diagnosing 'cervical weakness' in women with a history of preterm birth and/or second-trimester loss assist in the decision to place a history-indicated cerclage? | Green-top Guideline 60 – Cervical Cerclage | |
| **SV** | In women who have an incidental finding of a short cervical length, does amino acid analysis for infection guide who benefits from cervical cerclage?<br><br>[we need to construct this question] Does amino acid analysis for infection, in women with a short cervix, guide who benefits from cervical cerclage to prevent preterm birth? | Both (1), Service User (1) | |
| **SV** | Does pessary (progesterone?) help to prevent preterm birth? | Service User (1) HCP (1) | |
| **SR** | Should the decision on how best to minimise the risk of recurrent preterm birth in women at risk, either because of poor history of a short or dilated cervix, be 'personalised', based on the clinical circumstances, skill and expertise of the clinical team and, most importantly, the woman's informed choice? | Alfirevic (2011) | |

## 1.2.5 OTHER ANTENATAL INTERVENTIONS

| Source | Uncertainties | Reference | Notes |
|---|---|---|---|
| **SV** | Does treating fibroids reduce pre-term birth and if so what can be done to stop fibroids growing during pregnancy?<br>TWO QUESTIONS:<br>Is treating fibroids during pregnancy effective to reduce the risk of preterm birth?<br>What interventions are effective to prevent fibroids growing during pregnancy? | Service user (3) | |

| | | | |
|---|---|---|---|
| **SV, CG** | Does tocolysis lead to any benefit in preterm labour in multiple pregnancy?<br>Is maintenance tocolytic therapy following threatened preterm labour worthwhile?<br>TWO QUESTIONS:<br>Is tocolytic therapy effective to prevent preterm birth in multiple pregnancies?<br>In women threatening preterm labour is maintenance tocolytic therapy effective? | Green-top Guideline 1B – Tocolysis for Women in Preterm Labour<br>Smith V, Devane D, Begley C M; Clarke M, Higgins S (2009) A systematic review and quality assessment of systematic reviews of randomised trials of interventions for preventing and treating preterm birth. | |
| **CG** | What are the relative risks and benefits of delivery versus expectant management in women whose membranes have ruptured spontaneously between 34 and 37 weeks?<br>What are the risks and benefits of expectant management versus delivery in women whose membranes rupture spontaneously between 34 and 37 weeks gestation? | NICE clinical guideline 70 | |
| **SV, SR** | Which lifestyle changes including gym, bed rest, posture and sexual intercourse are effective to minimise the risk of preterm birth? (How do lifestyle changes impact preterm birth?) | Service User (2), Smith et al (2009) | |
| **SR** | Does magnesium maintenance therapy prevent preterm birth?<br>Is magnesium maintenance therapy effective to prevent preterm birth? | Han (2010) Magnesium maintenance therapy for preventing preterm birth after threatened preterm labour | |
| **SR** | What are the benefits and harms of immediate delivery compared with deferred delivery in cases of suspected fetal compromise at preterm gestations?<br>What are the benefits and harms of immediate versus deferred delivery of preterm infants with suspected fetal compromise? | Immediate versus deferred delivery of the preterm baby with suspected fetal compromise for improving outcomes | |

## 1.3 INTERVENTIONS AT BIRTH (PERINATAL)

### 1.3.1 PREMATURE RUPTURE OF MEMBRANES (PROM)

| Source | Uncertainties | Reference | Notes |
|---|---|---|---|
| **SV, CG** | How to predict latency interval (i.e PPROm) and delivery; treatments for Premature Rupture of Membranes (PROM)<br>TWO QUESTIONS:<br>CLINICAL HELP NEEDED WITH FIRST PART.<br>2ND PART: Which treatments are most effective for premature rupture of membranes? | Health care professional (2), service users (1), both (1) The Prevention of Early-onset Neonatal Group B Streptococcal Disease Green-top Guideline No. 36 | |
| **SR** | What are the clinical benefits and harms for women and their babies of immediate delivery compared with expectant management for women with preterm pre-labour rupture of the membranes? | Planned early birth versus expectant management for women with preterm prelabour rupture of membranes prior to 37 weeks' gestation for improving pregnancy outcome | |

## 1.3.2 CAESAREAN SECTION

| Source | Uncertainties | Reference | Notes |
|---|---|---|---|
| **SV** | Is delivery by caesarean section for late preterms necessary or beneficial?<br>Is delivery by caesarean section of benefit for late preterm infants? | Service user (1), both (1) | |

## 1.3.3 PROPHYLACTIC CORTICOSTEROID THERAPY IN PRETERM LABOUR (STEROIDS GIVEN BEFORE/DURING EARLY LABOUR) OR AFTER BIRTH

| Source | Uncertainties | Reference | Notes |
|---|---|---|---|
| **CG** | What are thelonger-term benefits and risks of multiple courses of antenatal corticosteroids? | Green-top Guideline 7 – Antenatal Corticosteroids to Reduce Neonatal Morbidity and Mortality | |

| | | | |
|---|---|---|---|
| | What are the long term benefits and risks of multiple courses of antenatal corticosteroids on neonatal morbidity and mortality? | | |
| **SV** | Long term follow-up of the effect of antenatal steroids on infant development<br>What are the long term effects of antenatal corticosteroids on infant development? | Health Care Professional (3), Both (1) | |

## 1.3.4 DELAYED CORD CLAMPING

| Source | Uncertainties | Reference | Notes |
|---|---|---|---|
| **SV** | When a baby is born very preterm, should the cord be clamped early or late?<br>Is early or late umbilical cord clamping, in very preterm births, more beneficial? | Both (2), HCP (1), service user (1) | |
| **SV, SR** | What is the optimal cord clamping timing?<br>What is the optimal umbilical cord clamping time for preterm infants? | Service User (1), HCP (1), Rabe (2012)<br>Rabe H, Reynolds G, Diaz-Rossello J (2008) *A systematic review and meta-analysis of a brief delay in clamping the umbilical cord of preterm infants*. Superceded by Rabe 2012 | |

## 1.3.5 OTHER PERINATAL INTERVENTIONS

| Source | Uncertainties | Reference | Notes |
|---|---|---|---|
| **SV** | Effectiveness of ante and intrapartum CTG monitoring for preterm infants.<br>Is antenatal and intrapartum cardiotocography beneficial to monitor preterm infants? | Health care professional (3) | |
| **SV** | Impact on fathers of preterm birth. For example, should fathers be invited to watch anything being done, or should they support their partners at the bedside? | Both (1) | |

| | | | |
|---|---|---|---|
| | What role should fathers play during the delivery of preterm infants? | | |
| **SV** | What makes a difference perinatally that will reduce the prevalence of subsequent disability or minimise the impact of potentially disabling conditions?<br>What is the best perinatal care for preterm infants to help reduce disabling conditions? | HCP (2) | |
| **SV** | What are the benefits of specialist antenatal care at preterm birth clinics for women at high risk of preterm birth?<br>KEEP ORIGINAL question | | |
| **SV** | Should extremely immature babies be intubated at delivery and surfactant given, or should CPAP be attempted? | Both (1)<br>HCP (1) | |

## 1.4 INTERVENTIONS AFTER BIRTH (POSTNATAL) UNTIL DISCHARGE FROM HOSPITAL

### 1.4.1 INITIAL CARE AND SUPPORT AT BIRTH

| Source | Uncertainties | Reference | Notes |
|---|---|---|---|
| **SV** | How do we improve resuscitation & supporting babies. | Health care professional (2), Both (1) | |
| **SV** | How can severe life threatening lung damage be best managed?<br>What is the best treatment for life threatening lung damage in preterm infants? | HCP(3), Service users (3) | |
| **SV** | The use of long-term ventilation on developmental outcomes for babies, any adverse effects? | HCP (4), Both (1), Service User (1) | |
| **SR** | Is High flow nasal cannula (HFNC) is safe or effective as a form of respiratory support in preterm infants? | Wilkinson (2011) **High flow nasal cannula for respiratory support** in preterm infants. | |

## 1.4.2 CARE AND FEEDING DURING HOSPITAL

| Source | Uncertainties | Reference | Notes |
|---|---|---|---|
| **SV** | What is the best way to encourage Kangaroo Mother Care more by staff in NICU for parents? | HCP (1) Service User (3) | |
| **SR** | Is community initiation of KMC beneficial? | Kangaroo mother care' to prevent neonatal deaths due to preterm birth complications | |
| **SR** | How effective is the use of early onset continuous KMC in stabilized LBW infants as an alternative to conventional neonatal care in resource limited settings? | **Conde-Agudelo (2011) Kangaroo mother care** to reduce morbidity and mortality in low birthweight infants | |
| **SV** | How can mothers be supported in breastfeeding their premature babies in NICU/SCBU/feeding clinics? | Service user (6), HCP (2), both (3) | |
| **SV** | Relationship regarding the impact/length of orogastric/nasogastric feeding and reflux on early feeding development and any other factors<br>What is the impact of length of orogastric / nasogastric feeding and reflux on early feeding development in preterm infants? | Health care professional (1), service user (1) | |
| **SR** | Does ad libitum or demand/semi-demand feeding regimen (versus scheduled interval feeding) affect important clinical outcomes for preterm infants and their families? | McCormick (2010) Ad libitum or demand/semi-demand feeding versus scheduled interval feeding for preterm infants | |
| **SV** | Optimise milk feeding how fast, how much, role of donor milk When to use formulas and which to use<br>What is the optimum milk feeding regimen, for preterm infants, including quantity and speed of feeding and use of donor and formula milks. | Both (2), HCP(3) | |
| **SR** | How does banked preterm milk versus banked term milk compare to promote growth and development in very low birth weight infants?<br>KEEP AS ORIGINAL QUESTION. | Dempsey (2010) Banked preterm versus banked term human milk to promote growth and development in very low birth weight infants | |

| | | | |
|---|---|---|---|
| **SV** | Providing clarity, knowledge and advice on breast milk vs formula for preterms. For example, at what point is it safe to introduce milk (formula milk) into a preterm diet when expressed breast milk is unavailable.<br>At what time is it safest to introduce formula milk to preterm infants when expressed breast milk is unavailable? | Health care professional (1), Service User (1), Both (1) | |
| **SR** | Does slow advancement of enteral feed volumes help to prevent necrotising enterocolitis in very low birth weight infants? | Morgan (2011) **Slow advancement of enteral feed volumes** to prevent neCGotising enterocolitis in very low birth weight infants. | |
| **SV** | How do we get quicker diagnosis of feeding problems including allergies. Failure to thrive as a result of poor feeding.<br>What is the best method to diagnose feeding problems in preterm infants, including allergies and failure to thrive due to poor feeding? | Service user(2), HCP (1) | |
| **SR** | What are the effects of oral motor interventions on pulmonary function compared to other interventions such as managing flow rate, providing pacing during oral feedings, and thickening of feedings?<br>What are the effects of oral motor interventions versus interventions such as managing flow rate, pacing during oral feeds and thickening of feedings, on pulmonary function in preterm infants? | Arvedson (2010) Evidence-based systematic review: effects of oral motor interventions on feeding and swallowing in preterm infants (American journal of speech-language pathology / American Speech-Language-Hearing Association) | |
| **SV** | Feeding difficulties in preterm infants and the long term effect on nutrition, growth and oro-motor development.<br>What are the long term effects, including nutrition, growth and oro –motor development, of feeding difficulties in preterm infants? | Health care professional (2) | |
| **SV** | What is the usefulness of breast milk fortifier How useful is breast milk fortifier?<br>Is breast milk fortifier beneficial for preterm infants? | HCP(1), Service user (1) | |
| **SV** | establishing oral feeding in preterm infants<br>How is it best to establish oral feeding in preterm infants? | HCP (2) | |
| **SV** | What are causes and treatments of NEC in preterm babies? Eg early decompressive surgery or watch and wait<br>TWO QUESTIONS:<br>What causes necrotising enterocolitis in preterm infants? | Service User (4), HCP (3) | |

| | | | |
|---|---|---|---|
| | Which treatments, including early de-compressive surgery and expectant management are most effective for necrotising enterocolitis in preterm infants? | | |
| **SV, SR** | What are the best way for neuro-developmental care and parenting -working towards a better quality of life and what is effectiveness of Newborn Individualized Developmental Care and Assessment Program (NIDCAP)?<br>TWO QUESTIONS:<br>Can neuro- development care and parenting influence a better quality of life for preterm infants?<br>Is the newborn individualised development care and assessment program (NIDCAP) effective to improve the quality of care of preterm infants? | Gaspardo (2010), Symington A, Pinelli J (2006) | |
| **SR** | What is the evidence to support the routine use of ethamsylate in preterm infants as a therapy for improving mortality or neurodevelopmental outcome?<br>Is routine use of ethamsylate effective to improve neuro-development and mortality in preterm infants? | Hunt (2010) Ethamsylate for the prevention of morbidity and mortality in preterm or very low birth weight infants | |

## 1.4.5 BONDING AND ATTACHMENT OF PARENTS AND INFANTS

| Source | Uncertainties | Reference | Notes |
|---|---|---|---|
| **SV** | What are the (long term) impact on attachment and bonding in parents with pre term infants? | Both (4), service users (3) | |

## 1.4.6 EFFECTIVE COMMUNICATION/SUPPORT TO PARENTS

| Source | Uncertainties | Reference | Notes |
|---|---|---|---|
| **SV** | What emotional or practical support services are available for parents and families of pre-term babies and is there any evidence to say what works better? | Service user (27), HCP(15), Both (2), | |

| | | | |
|---|---|---|---|
| | How can support be offered at all stages in the pre-term birth process? For example, food/accommodation facilities for parents during NICU stay, psychosocial interventions and stress reduction techniques | | |

## 1.4.7 SENSORY ISSUES

| Source | Uncertainties | Reference | Notes |
|---|---|---|---|
| **SV** | What is the impact of sensory care (eg sensory stimulation) or other factors (eg darkness of the room, times to sleep etc) during NICU stay of preterm infants on long term development (eg autistic disorder)? | Health care professional (5), Service user (3), Both (1) | |
| **SV** | Using ear muffs for developmental care. Also when using eye masks in very preterm for light protection, not just when under phototherapy<br>Does the use of ear muffs and eye masks on preterm infants support their developmental care? | Health Care Professional (2) | |
| **SR** | Is cycled light in the intensive care unit preferable to continuous bright light and to near darkness for preterm and low birth weight infants? | Morag (2011) Cycled light in the intensive care unit for preterm and low birth weight infants. | |
| **SV** | Is music therapy useful in NICU?<br>Is music therapy effective to support the wellbeing of preterm infants in the neonatal intensive care unit? | HCP (1), Service User (1) | |

## 1.4.8 PAIN MANAGEMENT OF INFANTS

| Source | Uncertainties | Reference | Notes |
|---|---|---|---|
| **SV** | What are the consequences of the routine use of morphine or ketamine for ventilated infants? | Health care professional (4) | |
| **SR** | What are the outcomes after long-term use of sucrose during painful procedures for very preterm and sick infants? What is the effectiveness of concomitantly administered sweet solutions and opioid analgesics, and effectiveness during longer procedures | Harrison (2010) Analgesic effects of sweet-tasting solutions for infants: current state of equipoise (Pediatrics), Stevens (2010) | |

| | | | |
|---|---|---|---|
| | | | |
| **SV, SR** | What do we know about pain perception/management/consequences of preterm babies? Eg Use of analgesia during intubations<br>Are interventions, including analgesia, effective for pain management in preterm infants? | Health care professional (3), Stevens (2010) | |
| **SR** | Which non-pharmacological measures relieve pain in the vulnerable group of sick and ventilated preterms?<br>(Non-pharmacological interventions = Non-nutritive and nutritive sucking, Music, Facilitated tucking, Swaddling, Positioning, Olfactory and multisensorial stimulation, Kangaroo care and maternal touch) | Cignacco Eva, Hamers Jan P H P; Stoffel Lilian, van Lingen Richard A A; Gessler Peter, McDougall Jane, Nelle Mathias (2006) The efficacy of non-pharmacological interventions in the management of procedural pain in preterm and term neonates. A systematic literature review.. European journal of pain (London, England). 11(2): 139-52. Stevens (2010) | |
| **SR** | What is the relationship between validated pain measures and indicators of nociceptive brain activity?<br>In preterm infants, what is the relationship between validated pain measures and indicators of nociceptive brain activity? | Stevens (2010) SuCGose for analgesia in newborn infants undergoing painful procedures | |

## 1.4.9 OTHER POSTNATAL INTERVENTIONS DURING HOSPITAL STAY

| Code | Uncertainties | Reference | Notes |
|---|---|---|---|
| **SV** | 1. Do preterm babies have better outcomes if their parents have roomed in?<br>2. What is role of siblings in caring for their pre-term sibling in hospital?<br>3. Do parents of preterm infants benefit from an open approach to notes and ward rounds?<br><br>[SG would like to merge above 3 questions] | Service user (1),<br>Health care professional (1),<br>Unknown source (1) | |
| **SV** | Do preterm infants benefit from much of the testing they undergo? eg. Lumbar puncture<br><br>What is the benefit of diagnostic testing, including for lumbar puncture, for preterm infants? | Health care professional (1)<br>Service User (1) | |

| | | | |
|---|---|---|---|
| **SV** | What do we know about treatment for brain damage? Why some babies are compromised with PVL/severe IVH when others don't. Does transporting babes increase incidence of IVH? Is stem cell therapy in PVL effective? Total body hypothermia in mild HIE? | HCP(5), Service user(1) | |
| **SV** | How can infection in preterm infants be better prevented? | HCP(2), both (1). Service user(2) | |
| **CG** | 1. What is the clinical and cost effectiveness of intrapartum antibiotic prophylaxis using benzylpenicillin in women with preterm labour?<br>What is the clinical and cost effectiveness of intrapartum prophylactic antiobiotic benzylpenicillin to prevent early onset neonatal infection?<br><br>2. Which risk factors for early-onset neonatal infection, clinical symptoms and signs of infection, and laboratory investigations should be used to identify babies who should receive antibiotics?<br>What are the best methods, including laboratory investigations, to identify risk factors and clinical signs and symptoms to identify babies needing antibiotics for early onset neonatal infection?<br><br>3. What is the clinical and cost effectiveness of laboratory investigations used individually or in combination to exclude early- onset neonatal infection in babies receiving antibiotics for suspected infection?<br>KEEP AS ORIGINAL QUESTION<br><br>4. What is the optimal duration of treatment (course length) in babies who receive antibiotics for confirmed early-onset neonatal infection?<br>What is the optimal duration of treatment in infants receiving antibiotics for confirmed early onset neonatal infections?<br><br>5. How does each step in the care pathway for prevention and treatment of early-onset neonatal infection impact on babies and their families?<br>KEEP AS ORIGINAL QUESTION<br><br>6. What is the clinical and cost-effectiveness of information and support offered to parents and carers of babies who have received antibiotics for | NICE clinical guideline 149 Antibiotics for early-onset neonatal infection | |

| | | | |
|---|---|---|---|
| | suspected or proven early-onset neonatal infection?<br>KEEP AS ORIGINAL QUESTION<br><br>7. Which risk factors for early-onset neonatal infection, clinical 5.2 symptoms and signs of infection, and laboratory investigations should be used to identify babies who should receive antibiotics? COULD COMBINE WITH 2 ABOVE.<br>➔ Can we combine above questions – DIFFICULT TO COMBINE WITHOUT LOOSING SPECIFICS OF INDIVIDUAL QUESTIONS | | |
| **CG** | Is drainage, irrigation and fibrinolytic therapy (DRIFT) an effective treatment in the management of post-haemorrhagic hydrocephalus in preterm infants who may suffer severe disability as a result? | NICE interventional procedure guidance 412 - Drainage, irrigation and fibrinolytic therapy (DRIFT) for post-haemorrhagic hydrocephalus in preterm infants | |
| **CG** | What is the comparative effectiveness and cost-effectiveness of universal pre-discharge transcutaneous bilirubin sCGeening alone or combined with a risk assessment in reducing jaundice-related neonatal morbidity and hospital readmission?<br>KEEP AS ORIGINAL QUESTION<br><br>What is the comparative accuracy of the Minolta JM-103 and the BiliChek when compared to serum bilirubin levels in all babies?<br>What is the accuracy of transcutaneous bilirubinometers, Minolta JM-103 and BiliCheck, indicated by total serum bilirubin levels in preterm infants?<br><br>How frequently and for how long can conventional phototherapy be interrupted without adversely effecting clinical outcomes?<br>How frequently and for how long can conventional phototherapy, for the treatment of neonatal jaundice, be interrupted without adversely effecting clinical outcomes?<br><br>What is the clinical and cost-effectiveness of LED phototherapy compared to conventional phototherapy in term and preterm babies with significant hyperbilirubinaemia? | Neonatal clinical guidance | |

| | | | |
|---|---|---|---|
| | KEEP AS ORIGINAL QUESTION<br><br>What is the clinical and cost-effectiveness of fibreoptic phototherapy using large pads compared to conventional phototherapy in *term* babies with significant hyperbilirubinaemia?<br>KEEP AS ORIGINAL QUESTION BUT SHOULD IT READ IN *PRETERM* BABIES?<br><br>What is the effectiveness, cost-effectiveness and safety of Clofibrate alongside phototherapy versus phototherapy alone for non-haemolytic significant hyperbilirubinaemia?<br>What is the effectiveness, cost-effectiveness and safety of Clofibrate alongside phototherapy versus phototherapy alone for non-haemolytic significant hyperbilirubinaemia in preterm infants?<br><br>What is the clinical and cost-effectiveness of IVIG when used to prevent exchange transfusion in newborns with haemolytic disease and rising bilirubin?<br>What is the clinical and cost effectiveness of intravenous immunoglobulin to prevent exchange transfusion in preterm newborns with haemolytic disease and rising bilirubin?<br><br>Can we combine above questions? DIFFICULT TO COMBINE. | | |

## 1.5 INTERVENTIONS AFTER BIRTH (POSTNATAL) AFTER DISCHARGE FROM HOSPITAL

### 1.5.1 END OF LIFE CARE

| Source | Uncertainties | Reference | Notes |
|---|---|---|---|
| **SV** | What is the best way to support parents with (1) decision-making in terms of stopping care and (2) following the death of their babies on the neo natal unit? | Both (1), Service User (1), hcp (3) | |
| **SV** | What support is required when babies are discharged?<br>Following-up after discharge? | Service User (3) | |

## 1.5.2 DURING TRANSITION

| Source | Uncertainties | Reference | Notes |
|---|---|---|---|
| **SR** | What is the effect on long-term growth and development of feeding preterm infants following hospital discharge with multi-nutrient fortified breast milk compared with unfortified breast milk? Which nutrient groups confer the most important benefits to growth and development?<br><br>TWO QUESTIONS:<br><br>Is multi-nutrient fortified breast milk compared with unfortified breast milk more effective for long term growth and development of preterm infants following hospital discharge?<br><br>Which nutrients are most effective for growth and development of preterm infants? | McCormick (2010) Multinutrient fortification of human breast milk for preterm infants following hospital discharge | |

## 1.6 GENERAL / PATIENT CARE

| Source | Uncertainties | Reference | Notes |
|---|---|---|---|
| **SV** | Examining the UK neonatal care model, consider change to Swedish model<br>Is the UK neonatal care model compared to the Swedish care model more effective for the care of preterm infants? | Both (2) | |
| **SV** | What is the effect of medical staff and parents working together and being inclusiv?<br>What is the effect of partnership working between healthcare professionals and parents on the care of preterm infants? | Service User (1) | |

## 1.7 STAFF ISSUES –

| Source | Uncertainties | Profile + No. of submissions | Notes |
|---|---|---|---|
| **SV** | What should healthcare professionals know about preterm birth (staff training about preterm birth)? | Service User (1),Health Care Professional (3) | |
| **SV** | What are adequate or additional trainings about preterm birth to midwives? | Service User (2) | |
| **SV** | Can we have support groups for healthcare professionals? | Service user (1), HCP (1) | |
| **SV** | What should the role of the specialist neonatal nurse be in the team in order to achieve the best long term developmental outcomes for infants and their families in neonatal care? | Health care professional (2) | |
