# Supplementary material for: Top research priorities for preterm birth: results of a prioritisation partnership between people affected by preterm birth and healthcare professionals
Source: BMC Pregnancy Childbirth. 2019 Dec 30;19:528. doi: 10.1186/s12884-019-2654-3 (PMC6938013; doi:10.1186/s12884-019-2654-3)
Supplement: Supplementary file 6 — Additional file 6. Reasons for excluding submissions. [file 12884_2019_2654_MOESM6_ESM.pdf]

Reasons for excluding submissions

Duplicates and excluded submissions based on discussion from Friday 8th March Steering Group meeting

Below is a list of excluded submissions based on discussion at the SG meeting.

(Red -> Reasons for exclusion)

Exclusion Criteria

- Duplicate
- Non-Intervention
- Not uncertainties
- Pre-pregnancy
- Post-discharge from hospital or long-term health condition
- Staffing Issues?

Pre-pregnancy Education

ID number	Original submissions	No. of submissions	ID numbers
1860238343	Are we getting any better at informing our young people about the causes of premature birth as a preventative measure. PRE-PREGNANCY	Health care professional (3)	

Treatment with Medications

ID number	Original submissions	No. of submissions	ID numbers
1849744543/1c	The effects of any illicit substance/ medication ie diazepam, on the foetus and length of withdrawal period thereafter NOT UNCERTAINTIES	HCP (1)	**Not uncertainties**
1837904252b	psychiatric drug affect on in utero babies that are born premature. NOT UNCERTAINTIES	Service user (1)	**Not uncertainties**
1850073716/d	how aspirin can work in pregnancy for those at risk etc NOT UNCERTAINTIES	Service user(1)	**Not uncertainties**

1849744543/1b	The effects of 'excessive' maternal caffeine intake on the infant in utero/ in breast milk<br>NOT UNCERTAINTIES	Health care professional(1)	**Not uncertainties**

Education during pregnancy

ID number	Original submissions	No. of submissions
1811391066/2	I also feel woman need to be given more information about (if it is possible)<br>how to help prevent premature labour/<br>birth.<br><br>PRE-PREGNANCY	Service user (1)

Other antenatal interventions

ID number	Original submissions	No. of submissions
1828052501/d	Are there non-invasive ways that could replace current invasive testing for some conditions?<br>NOT DIRECTLY RELATED TO PRETERM BIRTH	Health care professional (1)
1845609676	Platelets levels: safe level for physiotherapy interventions<br>INTERVENTION POST-DELIVERY	Health care professional (1)
1829354898/a	Can preterm birth be delayed in any medical way?<br>TOO BROAD	Service user (1)

Thermal care (keeping babies at the right temperature)

ID number	Original submissions	No. of submissions	ID numbers
1823560296/5	Does it matter if babies are allowed to get cold at delivery?<br>Duplicate, already answered	Both (1)	*There is one other submission on this*

Effective Communication/Support to parents

ID number	Original submissions	No. of submissions	Additional ID numbers
1951091908/2	How to consider the needs of all the family during preterm<br>DUPLICATE	1860226617/3	**See original document, a lot of submissions for this**
1813978902/2	Couselling/Reassurance provided – feedback of this service<br>DUPLICATE		
	Family support<br>DUPLICATE (in a number of sections)		

Other perinatal interventions

ID number	Original submissions	No. of submissions

1838009156/c	What makes a difference perinatally that will reduce the prevalence of subsequent disability or minimise the impact of potentially disabling conditions?<br>Too broad to be included	Health care professional(1)

Other interventions after discharge

ID number	Original submissions	No. of submissions	Additional ID numbers
1829443632	Access to services in the community for preterm babies?<br>POST-DISCHARGE (combine the following three items to 'what is the best way to look after babies and families after discharge?')	Both (1)	
1813978902/2	More home treatment to be available<br>POST-DISCHARGE	care at home (1)	
1811160846b	Family support after discharge incl access to services in the community<br>POST-DISCHARGE	Service user (2), both (1)	
P024	What can mothers do for PTB as they are growing up to improve development?<br>POST-DISCHARGE (combine the following four items)	Service User (1)	
P003	What about cognitive development therapy? Ways to support brain development eg music therapy POST-DISCHARGE	Service User (1)	
1925109617	Does responsive sensitive parenting result in improved long term developmental outcomes?<br>POST-DISCHARGE	HCP (2)	1895288557/1 (HCP)
1901490271b	Comfort holding, does the person comforting make a big difference?<br>POST-DISCHARGE	No input (1)	
P010/2	Pros & Cons of offering a pre-term birth clinic POST-DISCHARGE (combine the following three items to 'best way to debrief after preterm birth and prepare for future pregnancies')	Health Care Professional (1)	
P035	Thorough medical examinations after miscarriages to include causes OUT OF SCOPE	Service User (1)	
P008/2	Appropriate feedback of possible causes of PTB<br>DUPLICATE	Health Care Professional (1)	
P006/3	Role of older siblings in caring for their pre-term sibling<br><br>POST-DISCHARGE	Health care professional (1)	

1.8 Staff Issues

ID number	Original submissions	No. of submissions	Additional ID numbers
1949597774b	Staff in antenatal/labour wards capable of detecting labour starting and being able to take steps to stop labour progressing	Service User (1)	

	STAFF ISSUES<br>This is a staff education issue (a) what should staff know (b) how should staff be talking		
1949452693	The effects on medical staff and parents of parental presence at rounds and ways to develop policies so both groups maximise the benefits. Identifying weak points in the care cycle... do weekends and overnights affect levels of care especially in terms of continuity? Handovers: identify best practice to ensure SHOs and registrars ensure continuity of care. What support from the consultancy team works best? Communication with parents: do parents who receive regular communications (both written and verbal) feel better prepared and supported during the hospital stay? What information is most important?<br>STAFF ISSUES<br>Two themes (a) How should staff communicate about preterm babies with each other and with staff (b) what is the best pattern of care for preterms?	Service User (1)	
